# A molecular survey of orthohantaviruses in rodents across the tri-border region of China, Russia, and North Korea

**DOI:** 10.1371/journal.pntd.0014134

**Published:** 2026-04-20

**Authors:** Yunzhi Peng, Yiru Gu, Liang Li, Ge Zhang, Ziyan Liu, Ning Liu, Zhanli Wang, Jianting Xu, Zedong Wang

**Affiliations:** 1 Department of Infectious Diseases, Center of Infectious Diseases and Pathogen Biology, State Key Laboratory of Zoonotic Diseases, The First Hospital of Jilin University, Changchun, China; 2 State Key Laboratory of Pathogen and Biosecurity, Chinese Academy of Agricultural Sciences Changchun Veterinary Research Institute, Changchun, China; 3 Inner Mongolia Key Laboratory of Disease-Related Biomarkers, The Second Affiliated Hospital of Baotou Medical College, Baotou, China; 4 Cancer Center, The First Hospital of Jilin University, Changchun, China; 5 International Center of Future Science, Jilin University, Changchun, China; University of Tennessee College of Medicine: The University of Tennessee Health Science Center College of Medicine, UNITED STATES OF AMERICA

## Abstract

**Background:**

Hemorrhagic fever with renal syndrome (HFRS) is highly prevalent in northeastern China, especially along the borders with Russia and North Korea. However, the prevalence of HFRS-causing orthohantaviruses in rodents in these regions remains unclear.

**Methods:**

Rodents were captured across the tri-border region. Lung tissues were collected to detect human-pathogenic Hantaan virus (HTNV), Seoul virus (SEOV), and Amur virus (AMRV). Complete viral genome sequences from positive samples were amplified for phylogenetic and homology analyses.

**Results:**

A total of 430 rodents from four species were captured from five villages in the tri-border region, with *Apodemus agrarius* as the dominant species, accounting for 41.4%. HTNV was detected in 8.4% of *A. agrarius* and 3.6% of *R. norvegicus* individuals, with viral loads ranging from 2.2–6.9 log₁₀ copies/μL and 4.1–6.9 log₁₀ copies/μL, respectively. AMRV was identified in 6.3% of *A. peninsulae* individuals, with viral loads between 3.6–6.7 log₁₀ copies/μL. SEOV was not detected in any samples. No significant differences in viral prevalence were observed across years, seasons, or collection habitats, nor in viral load between virus species or rodent species. One AMRV and ten HTNV complete genome sequences were successfully obtained. Phylogenetic analysis revealed that the identified HTNV strains formed a distinct clade with previously reported strains from northeastern China and Russia, showing nucleotide and amino acid similarities of 87.5–97.6% and 97.1–100.0%, respectively. AMRV exhibited a close evolutionary relationship with strains isolated from patients and from *A. peninsulae* in China, displaying nucleotide and amino acid similarities of 80.6–84.7% and 92.3–97.7%, respectively, compared to HTNV.

**Conclusions:**

Our study confirms natural infections of HTNV in *A. agrarius* and *R. norvegicus*, and of AMRV in *A. peninsulae*, in the tri-border region of China, Russia, and North Korea. Further epidemiological studies on orthohantavirus infections in humans and rodents are warranted in this border region.

## Author summary

HFRS is highly prevalent in northeastern China, particularly in areas bordering Russia and North Korea. However, knowledge of orthohantavirus infection in rodent populations and the genetic diversity of circulating strains in this region remains limited. To address this gap, we conducted a molecular survey of orthohantaviruses in rodents across the China–Russia–North Korea tri-border region. From 2022 to 2023, 430 rodents were captured, with *A. agrarius* as the dominant species (41.4%). HTNV was detected in 8.4% of *A. agrarius* and 3.6% of *R. norvegicus*, while AMRV was identified in 6.3% of *A. peninsulae*. No SEOV was detected in any sample. Phylogenetic analysis showed that the HTNV strains formed a distinct clade with previously reported strains from northeastern China and Russia, whereas AMRV clustered closely with previous identified strains. These findings highlight the need for further epidemiological investigations into HTNV and AMRV infections in humans within this border area. The results provide valuable data for HFRS surveillance and control efforts in northeastern China.

## Introduction

Orthohantaviruses, a group of rodent-borne viruses belonging to the family *Hantaviridae*, are causative agents of Hemorrhagic fever with renal syndrome (HFRS) in Eurasia and Hantavirus pulmonary syndrome in the Americas [1]. In Eurasian countries, thousands of HFRS cases are reported annually, typically characterized by clinical symptoms such as fever, headache, hemorrhage, acute renal failure, and even death [2,3]. China has the highest incidence of HFRS, accounting for 90% of global cases, with nearly half occurring in northeastern China [4,5]. In particular, the eastern prefecture-level cities in northeastern China that border Russia or North Korea are at high risk for HFRS epidemics [6].

Hunchun City, located in the Yanbian Korean Autonomous Prefecture of Jilin Province in northeastern China, is the only Chinese border area situated at the trijunction of China, Russia, and North Korea, sharing mountainous terrain and riverine boundaries with both neighboring countries. In this region, rodents and other small mammals can easily cross borders, spreading the pathogens they carry. As a national-level HFRS surveillance site, Hunchun has documented infections of Hantaan virus (HTNV) and Seoul virus (SEOV) in both rodents and humans [7–9]; these viruses are the two most common causes of HFRS in northeastern China and are primarily carried by *Rattus norvegicus*, *Apodemus peninsulae*, and *Apodemus agrarius* [10].

**Fig 1:**
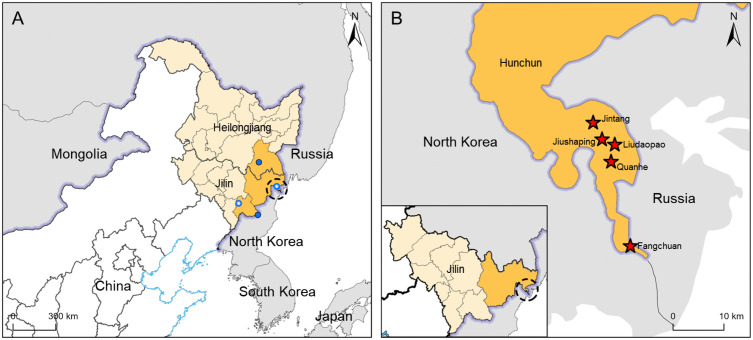
Geographical map and rodent sample collection in the tri-border region of China, Russia, and North Korea. **(A)** Geographical map of the tri-border region of China, Russia, and North Korea. Amur virus distribution in northeastern China from previous studies is shown with dots. Solid blue dots indicate sites with precise coordinates; hollow blue circles indicate sites with imprecise geographical information. **(B)** Rodent sample collection in the tri-border region. The red stars indicate the sample collection sites. Map Approval Numbers: GS(2016)1666 and GS(2023)2767.

Another causative agent of HFRS, Amur virus (AMRV), along with HTNV, belongs to the *Orthohantavirus hantanense* species [11]. In China, AMRV has been confirmed to be distributed around the Changbai Mountains, with detections reported in humans, *A. peninsulae*, and *Myodes rufocanus* from Mudanjiang City, Heilongjiang Province, and in *A. peninsulae* from Changbai, Hunchun, and Jingyu counties, Jilin Province (Fig 1A) [12–15].

Given that Hunchun and its surrounding areas serve as natural reservoirs for HTNV, SEOV, and AMRV, and that spillover events involving these viruses commonly occur among different rodent species, this study aims to conduct molecular surveillance of these orthohantaviruses in rodents along the China–North Korea–Russia tri-border region to clarify their prevalence in rodent populations, thereby providing reliable data for HFRS prevention and control in border regions.

## Materials and methods

### Ethics statement

The animal studies were approved by the Animal Administration and Ethics Committees of the Changchun Veterinary Research Institute, Chinese Academy of Agricultural Sciences (Approval numbers: IACUC of AMMS -11-2020-026). Rodents were caught alive and handled strictly in accordance with the ethical guidelines for the care and use of experimental animals issued by the Ministry of Science and Technology of China.

### Collection of rodent samples

Rodents were captured during the spring and fall seasons from 2022 to 2023 using a nocturnal trapping method in the tri-border region of China, Russia, and North Korea. A total of 100 live traps were deployed at 5-meter intervals and baited with peanuts over seven consecutive days during each trapping period. Sampling sites included residential areas, farmlands, wetlands, and forested areas in five villages: Jintang (42.66°N, 130.52°E), Jiushaping (42.63°N, 130.54°E), Liudaopao (42.62°N, 130.57°E), Quanhe (42.59°N, 130.56°E), and Fangchuan (42.44°N, 130.60°E).

The sample size was not determined using statistical methods, as this would require baseline data on orthohantavirus prevalence, which were not available at the study design stage. Animal dissections were performed under ether anesthesia, and all appropriate measures were implemented to minimize animal distress. Lung tissues were collected and immediately placed in portable cooling units containing dry ice, then transferred to -80°C freezers for long-term storage. Rodent species identification was initially confirmed based on morphological characteristics, and representative individuals were later confirmed by sequencing and analyzing the partial cytochrome C oxidase (COI) gene as previously reported [16].

### Detection and quantification of orthohantaviruses in rodent samples

Viral RNA was extracted from the lung tissues using the TIANamp Virus RNA Kit (TIANGEN, China), and reverse-transcribed into complementary DNA using the PrimeScript 1st Strand cDNA Synthesis Kit (TaKaRa, Japan) according to the manufacturer’s instructions. Specific primers and probes targeting the conserved regions of the S segment of HTNV and AMRV, and the L segment of SEOV, were designed based on viral strains identified in northeastern China, the Russian Far East, and South Korea to detect the viruses (S1 Table).

TaqMan probe-based reverse transcription quantitative real-time polymerase chain reaction (RT-qPCR) assays were developed and evaluated using HTNV, AMRV, and SEOV positive human and rodent samples (S1–S3 Figs). The Ct values of virus-positive samples were converted into viral copies using the equations y = −3.240x + 45.926 for HTNV, y = −3.421x + 44.542 for AMRV, and y = −3.957x + 45.854 for SEOV, derived from the established RT-qPCR assays. A sample was classified as positive for the virus if it exhibited a Ct value below 40 in at least one replicate.

### Viral genome amplification

For positive samples, semi-nested PCR was conducted to amplify complete genome sequence using the primers listed in S2 Table. The PCR products were purified using a gel extraction kit (TIANGEN, China) and sequenced using the Sanger method. Full-genome amplification was discontinued in cases where (1) the viral titer in the samples was insufficient to support robust amplification, and (2) preliminary sequence analysis indicated exceptionally high inter-sample identity, suggesting limited genetic diversity.

### Phylogenetic and homology analyses

To analyze the phylogenetic and homology relationships among the strains identified in this study and other viral strains from humans and rodents in northeastern China, the Russian Far East, and South Korea, representative reference virus strains were downloaded from GenBank for analysis (S3 and S4 Tables).

The nucleotide sequences of the open reading frames were aligned using ClustalW implemented in MEGA 7.0. The GTR + G, GTR + G + I, and GTR + G + I models were selected as best-fit for L, M, and S segments, respectively, using the Find Best DNA/Protein Models (ML) plugin in MEGA 7.0, based on their lowest Bayesian Information Criterion (BIC) scores [17]. Phylogenetic trees were constructed using the Maximum Likelihood method with 1,000 bootstrap replicates, visualized in the traditional layout with a rectangular branch style. Branches with bootstrap support values greater than 70% were considered highly reliable and are indicated at the corresponding nodes. The scale bar represents the relative proportion of branch lengths, reflecting evolutionary distance differences among branches. Sequence identities were calculated using the MegAlign module in DNASTAR software and visualized as heatmaps generated by GraphPad Prism 8.

### Statistical analyses

The prevalence of the virus across different animal and viral species was analyzed using Pearson’s chi-square test or Fisher’s exact test, as appropriate. Viral titers were compared using the Mann-Whitney U test. A *p*-value < 0.05 was considered statistically significant. Statistical computations were conducted using GraphPad Prism software or SPSS 22.0 (SPSS Inc.).

## Results

### Collection of rodents

A total of 430 rodents were captured from five villages in the tri-border region (Fig 1 and Table 1). Among them, *A. agrarius* was the predominant species, accounting for 41.4% (178/430). Other species included *R. norvegicus* (n = 111), *A. peninsulae* (n = 79), *Cricetulus barabensis* (n = 41), and *Microtus fortis* (n = 21), accounting for 25.8%, 18.4%, 9.5%, and 4.9%, respectively (Table 1). Among them, 209 rodents were captured in 2022 and 221 in 2023; 199 were collected in spring and 231 in fall. Most of the rodents were collected from farmlands, accounting for 30.5% (131/430), while the remaining rodents were collected from residential areas, wetlands, and forested areas, representing 24.4% (n = 105), 18.8% (81), and 26.3% (113), respectively (Tables 1 and 2).

**Table 1:** Orthohantavirus infections by season in trapped rodents in the tri-border region*.

Year	Season	Species					Total
		*Cricetulus barabensis*	*Rattus norvegicus*	*Apodemus agrarius*	*Apodemus* *peninsulae*	*Microtus fortis*	
2022	Spring	11/0	32/1 (HTNV)	34/3 (HTNV)	8/0	8/0	93/4
	Autumn	10/0	20/0	58/5 (HTNV)	25/2 (AMRV)	3/0	116/7
2023	Spring	11/0	41/2 (HTNV)	33/3 (HTNV)	14/1 (AMRV)	7/0	106/6
	Autumn	9/0	18/1 (HTNV)	53/4 (HTNV)	32/2 (AMRV)	3/0	115/7
Total		41/0	111/4 (HTNV)	178/15 (HTNV)	79/5 (AMRV)	21/0	430/24

*Data showing the number of rodents trapped/number of RT-qPCR-positive (virus species).

**Table 2:** Orthohantavirus infections by habitats in trapped rodents in the tri-border region*.

Habitats	Species					Total
	*Cricetulus barabensis*	*Rattus norvegicus*	*Apodemus agrarius*	*Apodemus peninsulae*	*Microtus fortis*	
Residential area	0/0	96/3 (HTNV)	9/1 (HTNV)	0/0	0/0	105/4
Farmland	7/0	5/1 (HTNV)	116/9 (HTNV)	0/0	3/0	131/10
Wetland	23/0	7/0	33/2 (HTNV)	0/0	18/0	81/2
Forest	11/0	3/0	20/3 (HTNV)	79/5 (AMRV)	0/0	113/8
Total	41/0	111/4 (HTNV)	178/15 (HTNV)	79/5 (AMRV)	21/0	430/24

*Data showing the number of rodents trapped/number of RT-qPCR-positive (virus species).

### RT-qPCR detection of orthohantaviruses

**Fig 2:**
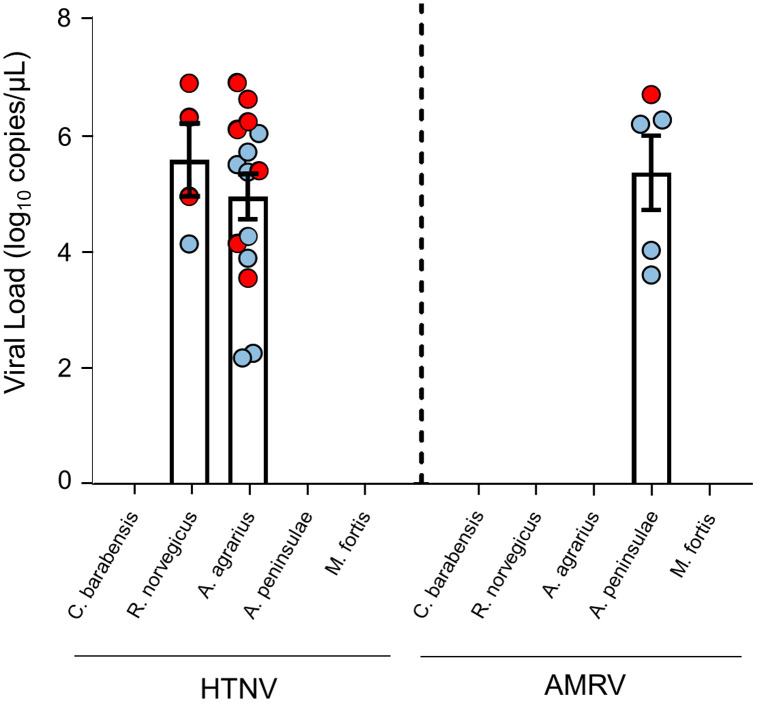
Viral load of Hantaan virus and Amur virus in lung tissues of rodents. The red dots indicate positive samples for which complete genome sequences were obtained.

Lung tissues from rodents were tested for HTNV, SEOV, and AMRV. HTNV was found in 15 *A. agrarius* (8.4%) and 4 *R. norvegicus* (3.6%) (Table 1); AMRV in 5 *A. peninsulae* (6.3%); SEOV in none. No significant difference in HTNV infection rate occurred between *A. agrarius* and *R. norvegicus* (*p* = 0.108) (S5 Table). HTNV viral loads were 2.2–6.9 log₁₀ copies/μL in *A. agrarius* and 4.1–6.9 in *R. norvegicus*; AMRV loads in *A. peninsulae* were 3.6–6.7 log₁₀ copies/μL. No significant difference in viral copies was observed between virus or rodent species (Fig 2 and S6 Table).

HTNV infection rates in *A. agrarius* and *R. norvegicus* were 9.0% (6/67) and 4.1% (3/73) in spring, and 8.1% (9/111) and 2.6% (1/38) in fall; AMRV infection rates in *A. peninsulae* were 4.5% (1/22) and 7.0% (4/57), respectively (Table 1). In residential area, farmland, wetland, and forest, HTNV infection rates in *A. agrarius* were 11.1% (1/9), 7.8% (9/116), 6.1% (2/33), and 15.0% (3/20); in *R. norvegicus*, 3.1% (3/96), 20.0% (1/5), 0.0% (0/7), and 0.0% (0/3); and AMRV infection rates in *A. peninsulae* were 0.0% (0/0), 0.0% (0/0), 0.0% (0/0), and 6.3% (5/79), respectively (Table 2). No significant difference was observed (*p* > 0.05) (S5 Table).

### Phylogenetic and homology analysis

**Fig 3:**
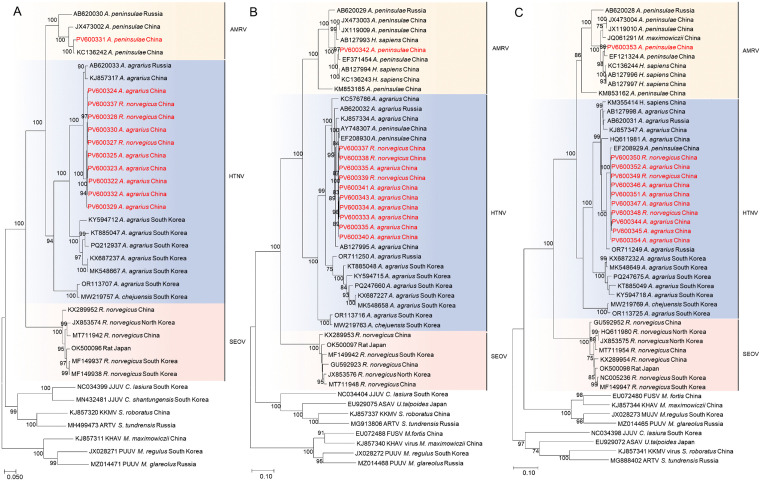
Phylogenetic analysis of the open reading frame nucleotide sequences from the L, M, and S segments of the identified viral strains. Strains identified in this study are highlighted in red. Bootstrap values >70% are shown at nodes and considered reliable. The scale bar represents branch lengths, reflecting evolutionary distances. Abbreviations: HTNV, Hantaan virus; AMRV, Amur virus; SEOV, Seoul virus; MUJV, Muju virus; JJUV, Jeju virus; ASAV, Asama virus; KKMV, Kenkeme virus; ARTV, Artybash virus; KHAV, Khabarovsk virus; PUUV, Puumala virus; FSNV, Fusong virus.

A total of one AMRV and ten HTNV (seven from *A. agrarius* and three from *R. norvegicus*) complete genome sequences were successfully amplified, with NCBI accession numbers listed in S7 Table. Partial viral sequences were obtained from the remaining 13 positive samples (S8 Table). Phylogenetic trees were constructed based on the open reading frame nucleotide sequences of the L, M, and S segments, incorporating both reference hantaviruses and the viral strains characterized in this study. The analysis revealed that the newly identified HTNV strains clustered together in a well-supported clade with previously reported HTNV strains from northeastern China, the Russian Far East, and South Korea. AMRV showed a close phylogenetic relationship with strains identified in patients and in *A. peninsulae* in China (Fig 3).

**Fig 4:**
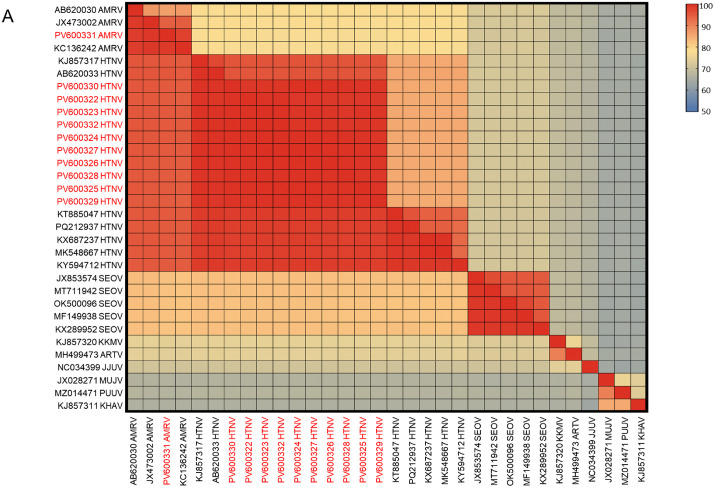
Homology analysis of the RNA-dependent RNA polymerase gene sequences of the identified Hantaan virus and Amur virus strains. Nucleotide sequence identities are shown in the upper-right quadrant of the heatmap, whereas amino acid sequence identities are shown in the lower-left quadrant. Strains identified in this study are highlighted in red. Abbreviations: HTNV, Hantaan virus; AMRV, Amur virus; SEOV, Seoul virus; KKMV, Kenkeme virus; ARTV, Artybash virus; JJUV, Jeju virus; MUJV, Muju virus; PUUV, Puumala virus; KHAV, Khabarovsk virus.

The identified HTNV strains showed 99.1–100.0% nucleotide and 99.4–100.0% amino acid homology among themselves (Figs 4 and S4 and S1–S3 Data). Compared to strains from northeastern China, South Korea, and Russia, they exhibited 87.5–97.6% nucleotide and 97.1–100.0% amino acid similarity. The AMRV strain shared 95.2–98.3% nucleotide and 99.0–99.5% amino acid homology with strains from patients and rodents in northeastern China, and showed 80.6–84.7% nucleotide and 92.3–97.7% amino acid similarity to HTNV (Figs 4 and S4 and S1–S3 Data).

## Discussion

We systematically conducted a molecular survey of HTNV, AMRV, and SEOV in rodents across the tri-border region of China, Russia, and North Korea, and identified HTNV in *A. agrarius* and *R. norvegicus*, as well as AMRV in *A. peninsulae*, in this area. These findings not only reflect the prevalence and genetic diversity of orthohantaviruses in the region of China, but also provide indirect evidence of their presence in neighboring areas of Russia and North Korea. This is particularly significant due to the limited epidemiological data currently available on these viruses in North Korea.

In this study, *A. agrarius* (41.4%) and *R. norvegicus* (25.8%) were the two most dominant species collected for detection (Tables 1 and 2). This is primarily because the rodents were captured in villages, farmlands, and wetland areas, which are the principal habitats of these two rodent species. Although *A. peninsulae* is common in forests, relatively fewer individuals were captured in this study, as most collection sites were at the forest–farmland interface. Deep forest access was restricted and hazardous due to the area’s designation as the Northeast China Tiger and Leopard National Park.

In northeastern China, HTNV causes the majority of HFRS cases, which are characterized by more severe clinical manifestations and a higher fatality rate compared to SEOV infection [18]. HTNV is primarily transmitted by *A. agrarius*, whereas SEOV is mainly maintained by *R. norvegicus*. Nevertheless, spillover infections—HTNV from *A. agrarius* to *R. norvegicus* and SEOV from *R. norvegicus* to *A. agrarius*—are relatively common in natural settings [19,20]. In this study, HTNV was detected in both *A. agrarius* and *R. norvegicus* (Table 1). Phylogenetic and homology analyses revealed that the HTNV strains from these two rodent species clustered within the same clade and shared nearly 100% sequence identity, indicating that this HTNV group can naturally infect *R. norvegicus* (Figs 3, 4 and S4).

Notably, no SEOV infection was detected in the collected rodents, neither in the primary host *R. norvegicus* nor in the spillover host *A. agrarius*. Given the adequate sample size and careful accounting for the living environments of captured rodents, potential confounding effects of regional variability or trapping bias on SEOV detection rates were ruled out. Although SEOV has been detected in Hunchun, no study has previously focused on this tri-border region [7,8]. We suggest that SEOV is either not prevalent or maintained at a consistently low positive rate in rodents in this area.

Given the high sequence identity and close phylogenetic relationship among AMRV, Soochong virus (SOOV), and HTNV, the International Committee on Taxonomy of Viruses (ICTV) has classified all three viruses within the species *Orthohantavirus hantanense* [11]. Since HTNV is known to infect both *A. agrarius* and *A. peninsulae*, we speculate that AMRV may also have the potential to infect *A. agrarius*. However, in this study, AMRV was detected only in *A. peninsulae*, consistent with previous reports [13–15].

Our study has several limitations. First, complete genome sequences were not obtained from all virus-positive samples, partly due to low viral titers that hindered genome amplification and partly because some partially sequenced isolates exhibited extremely high sequence identity with the already available complete genomes. Second, rodent monitoring in this area was conducted over a relatively short period and with a relatively small sample size in this study, which may account for the failure to detect *R. norvegicus* infected with SEOV.

## Conclusions

This study confirms natural infections of HTNV in *A. agrarius* and *R. norvegicus*, and of AMRV in *A. peninsulae*, in the tri-border region of China, Russia, and North Korea. Future studies should prioritize large-scale epidemiological investigations of orthohantavirus infections in humans and rodents in this region to support evidence-based prevention and control of HFRS.

## Supporting information

S1 Data Pairwise sequence identities of the L segment and RdRp among hantaviruses. Nucleotide sequence similarity of L segment (upper right) and amino acid sequence similarity of RdRp (lower left) of hantaviruses. (XLSX)

S2 Data Pairwise sequence identities of the M segment and glycoprotein precursor among hantaviruses. Nucleotide sequence similarity of M segment (upper right) and amino acid sequence similarity of the glycoprotein precursor (lower left) of hantaviruses. (XLSX)

S3 Data Pairwise sequence identities of the S segment and NP among hantaviruses. Nucleotide sequence similarity of S segment (upper right) and amino acid sequence similarity of NP (lower left) of hantaviruses. (XLSX)

S1 Fig Development of an RT-qPCR assay for HTNV. (A) Amplification plot for the detection of the HTNV nucleocapsid protein gene. (B) Standard curve for the detection of the HTNV nucleocapsid protein gene.
(DOCX)

S2 Fig Development of an RT-qPCR assay for AMRV. (A) Amplification plot for the detection of the AMRV nucleocapsid protein gene. (B) Standard curve for the detection of the AMRV nucleocapsid protein gene.
(DOCX)

S3 Fig Development of an RT-qPCR assay for SEOV. (A) Amplification plot for the detection of the SEOV RNA-dependent RNA polymerase gene. (B) Standard curve for the detection of the SEOV RNA-dependent RNA polymerase gene.
(DOCX)

S4 Fig Homology analysis of the glycoprotein precursor and nucleocapsid protein gene sequences of the identified Hantaan virus and Amur virus strains. Nucleotide sequence identities are shown in the upper-right quadrant of the heatmap, whereas amino acid sequence identities are shown in the lower-left quadrant. Strains identified in this study are highlighted in red. Abbreviations: HTNV, Hantaan virus; AMRV, Amur virus; SEOV, Seoul virus; KKMV, Kenkeme virus; ARTV, Artybash virus; JJUV, Jeju virus; MUJV, Muju virus; PUUV, Puumala virus; KHAV, Khabarovsk virus.
(DOCX)

S1 Table Primers and probes used in RT-qPCR assays for the detection of Hantaan virus, Amur virus, and Seoul virus. (DOCX)

S2 Table Primers used for genome amplification of Hantaan virus and Amur virus. (DOCX)

S3 Table Reference orthohantavirus strains used for phylogenetic analysis. (DOCX)

S4 Table Reference orthohantavirus strains used for homology analysis. (DOCX)

S5 Table Statistical analyses of the prevalence of Hantaan virus and Amur virus in rodents collected from the tri-border region of China, Russia, and North Korea. (DOCX)

S6 Table Statistical analyses of viral titers among different host and virus species. (DOCX)

S7 Table Information on the 11 complete genome sequences of the amplified viral strains in this study. (DOCX)

S8 Table Information on the remaining viral strains for which partial sequences were amplified in this study. (DOCX)
