# Supplementary material for: A molecular survey of orthohantaviruses in rodents across the tri-border region of China, Russia, and North Korea
Source: PLoS Negl Trop Dis. 2026 Apr 20;20(4):e0014134. doi: 10.1371/journal.pntd.0014134 (PMC13120696; doi:10.1371/journal.pntd.0014134)
Supplement: S1 Fig — (A) Amplification plot for the detection of the HTNV nucleocapsid protein gene. (B) Standard curve for the detection of the HTNV nucleocapsid protein gene. (DOCX) [file pntd.0014134.s004.docx]

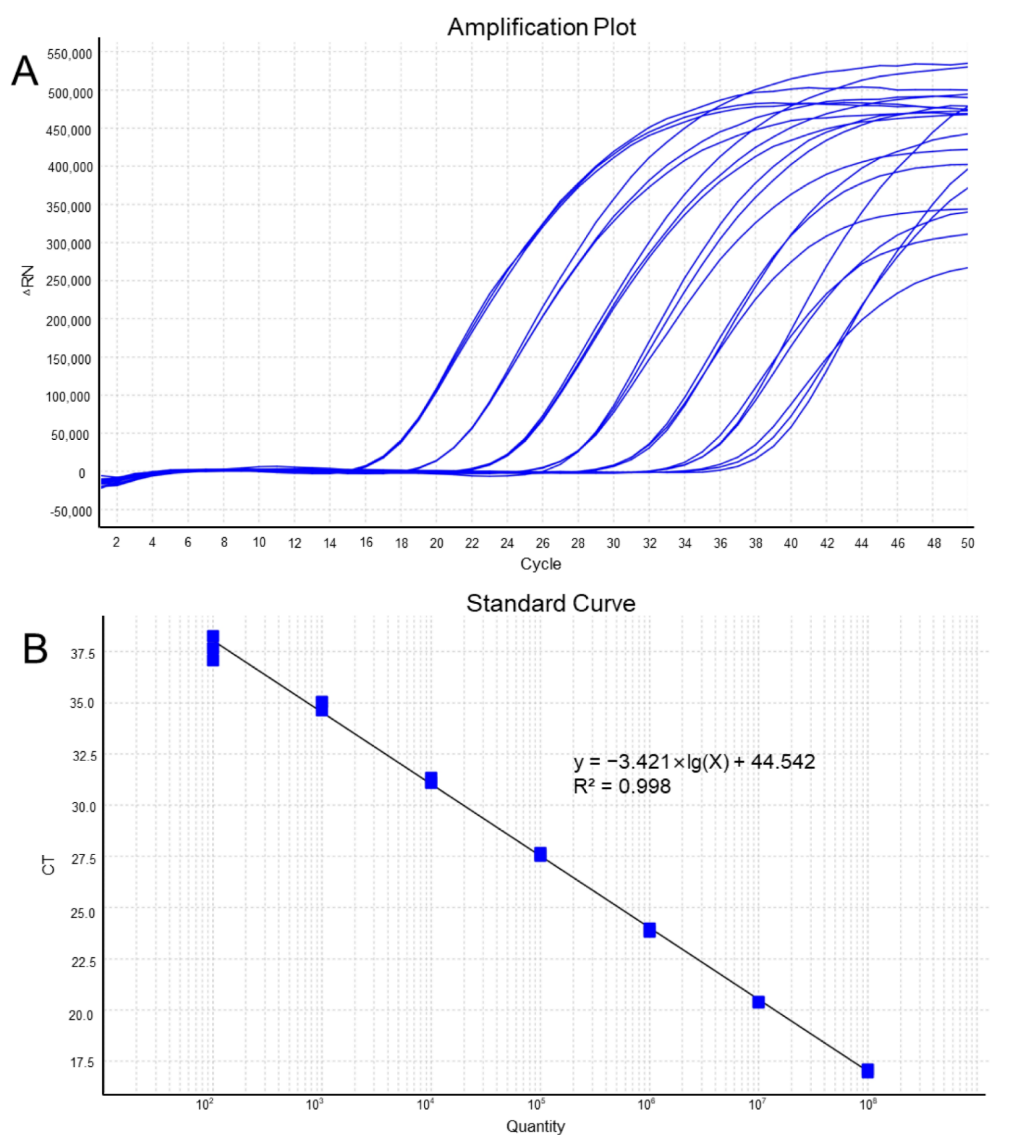


**S2 Fig.** Development of an RT-qPCR assay for AMRV. (A) Amplification plot for the detection of the AMRV nucleocapsid protein gene. (B) Standard curve for the detection of the AMRV nucleocapsid protein gene.
