# Supplementary material for: A molecular survey of orthohantaviruses in rodents across the tri-border region of China, Russia, and North Korea
Source: PLoS Negl Trop Dis. 2026 Apr 20;20(4):e0014134. doi: 10.1371/journal.pntd.0014134 (PMC13120696; doi:10.1371/journal.pntd.0014134)
Supplement: S2 Fig — (A) Amplification plot for the detection of the AMRV nucleocapsid protein gene. (B) Standard curve for the detection of the AMRV nucleocapsid protein gene. (DOCX) [file pntd.0014134.s005.docx]

**
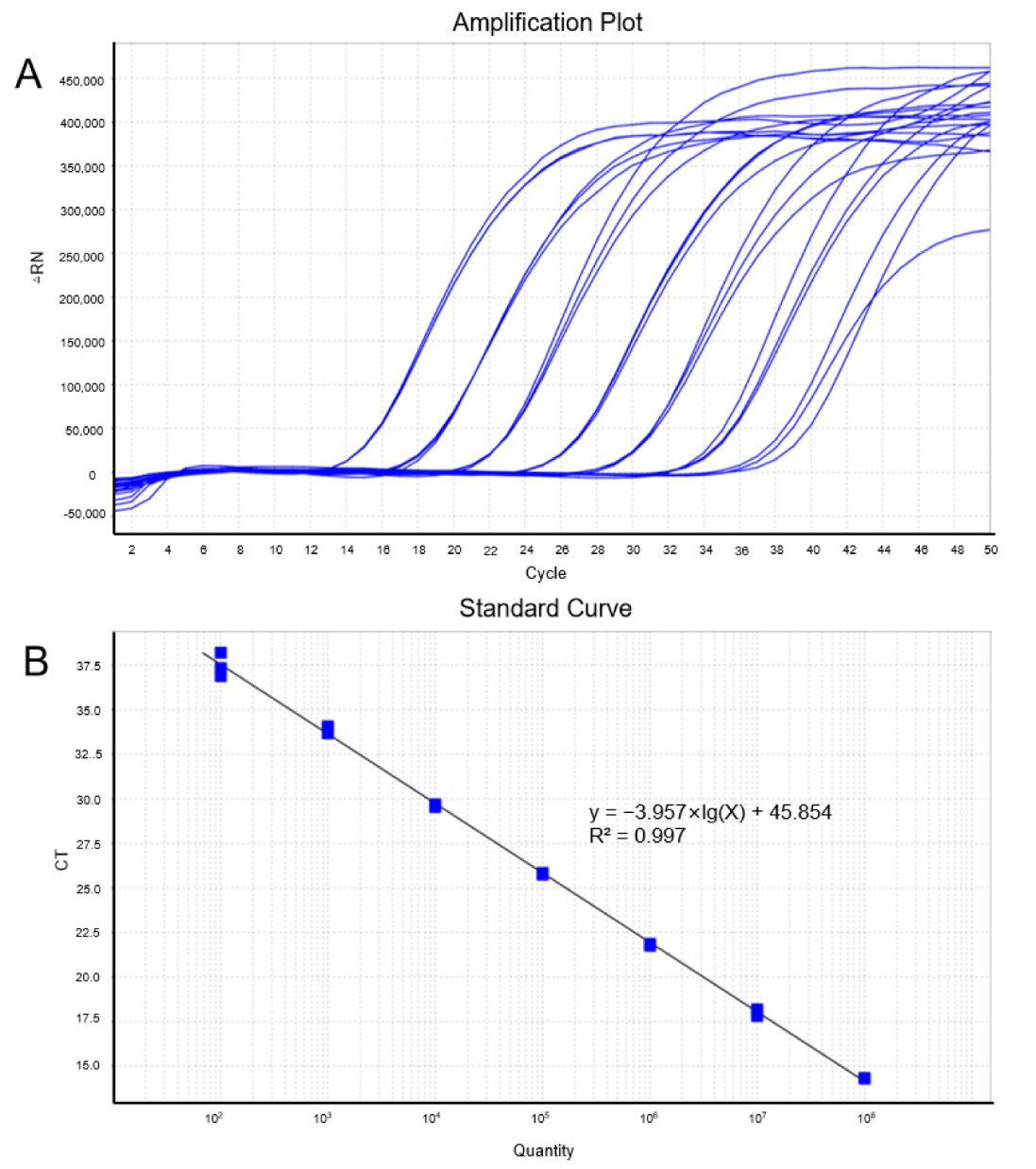
**

**S3 Fig.** Development of an RT-qPCR assay for SEOV. (A) Amplification plot for the detection of the SEOV RNA-dependent RNA polymerase gene. (B) Standard curve for the detection of the SEOV RNA-dependent RNA polymerase gene.
