# Supplementary material for: A molecular survey of orthohantaviruses in rodents across the tri-border region of China, Russia, and North Korea
Source: PLoS Negl Trop Dis. 2026 Apr 20;20(4):e0014134. doi: 10.1371/journal.pntd.0014134 (PMC13120696; doi:10.1371/journal.pntd.0014134)
Supplement: S3 Fig — (A) Amplification plot for the detection of the SEOV RNA-dependent RNA polymerase gene. (B) Standard curve for the detection of the SEOV RNA-dependent RNA polymerase gene. (DOCX) [file pntd.0014134.s006.docx]

**
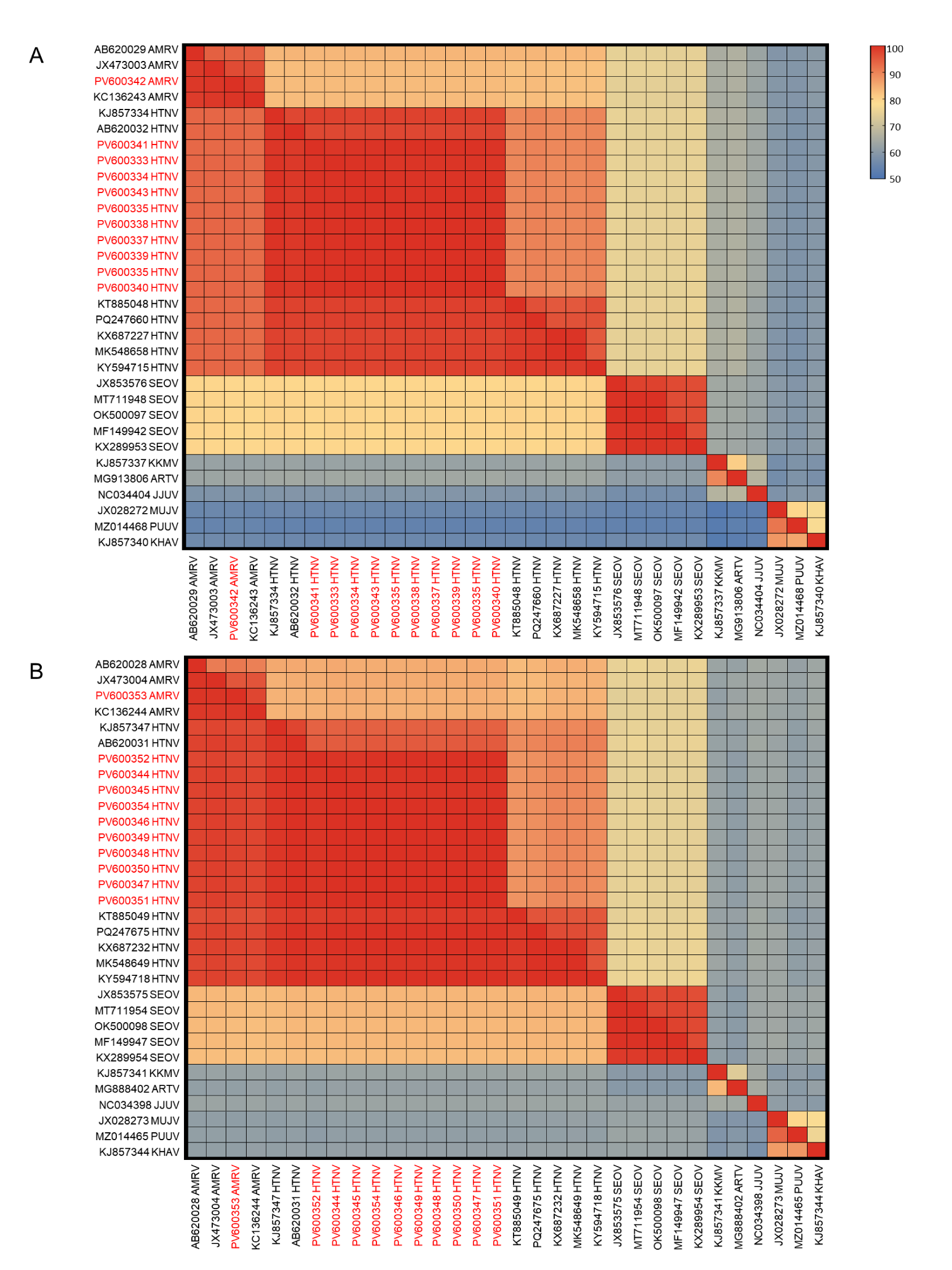
**

**S4 Fig.** Homology analysis of the glycoprotein precursor and nucleocapsid protein gene sequences of the identified Hantaan virus and Amur virus strains. Nucleotide sequence identities are shown in the upper-right quadrant of the heatmap, whereas amino acid sequence identities are shown in the lower-left quadrant. Strains identified in this study are highlighted in red. Abbreviations: HTNV, Hantaan virus; AMRV, Amur virus; SEOV, Seoul virus; KKMV, Kenkeme virus; ARTV, Artybash virus; JJUV, Jeju virus; MUJV, Muju virus; PUUV, Puumala virus; KHAV, Khabarovsk virus.
