# Supplementary material for: A molecular survey of orthohantaviruses in rodents across the tri-border region of China, Russia, and North Korea
Source: PLoS Negl Trop Dis. 2026 Apr 20;20(4):e0014134. doi: 10.1371/journal.pntd.0014134 (PMC13120696; doi:10.1371/journal.pntd.0014134)
Supplement: S4 Fig — Nucleotide sequence identities are shown in the upper-right quadrant of the heatmap, whereas amino acid sequence identities are shown in the lower-left quadrant. Strains identified in this study are highlighted in red. Abbreviations: HTNV, Hantaan virus; AMRV, Amur virus; SEOV, Seoul virus; KKMV, Kenkeme virus; ARTV, Artybash virus; JJUV, Jeju virus; MUJV, Muju virus; PUUV, Puumala virus; KHAV, Khabarovsk virus. (DOCX) [file pntd.0014134.s007.docx]

**S1 Table.** Primers and probes used in RT-qPCR assays for the detection of Hantaan virus, Amur virus, and Seoul virus.

| **Virus** | **Primers** | **Sequence (5' → 3')** |
| --- | --- | --- |
| Hantaan virus | HTNV-F^a^ | CCACCTGGATATTGATGA |
|  | HTNV-R^b^ | GGATGTAAGATAGACAACAATA |
|  | HTNV-P^c^ | FAM-CRACAGGACAGACAGCAGACT-BHQ1 |
|  |  |  |
| Amur virus | AMRV-F^a^ | AGAGCACTAACAGACAGG |
|  | AMRV-R^b^ | CCAGTACATTCCCATAGC |
|  | AMRV-P^c^ | FAM-AGTCGCAGCATCCATTCARGC-BHQ1 |
|  |  |  |
| Seoul virus | SEOV-F^a^ | CTCCAGGTGACAATTCAG |
|  | SEOV-R^b^ | GGTTCATAAGTATCCATAGAATC |
|  | SEOV-P^c^ | FAM-AGGCGATTCACTGCTGCTCT-BHQ1 |

*a. Forward; b. Reverse; c. Probe.
