# Supplementary material for: A molecular survey of orthohantaviruses in rodents across the tri-border region of China, Russia, and North Korea
Source: PLoS Negl Trop Dis. 2026 Apr 20;20(4):e0014134. doi: 10.1371/journal.pntd.0014134 (PMC13120696; doi:10.1371/journal.pntd.0014134)
Supplement: S1 Table — (DOCX) [file pntd.0014134.s008.docx]

**S2 Table.** Primers used for genome amplification of Hantaan virus and Amur virus.

| **Virus** | **Primers** | **Sequence (5' → 3')** | **Amplicon (bp)** |
| --- | --- | --- | --- |
| Hantaan virus | HTNV-S-1F | TAGTAGTAGACTCCCTAAAG | 425 |
|  | HTNV-S-440R1 | TTAACATATACAGAGCTTTWA |  |
|  | HTNV-S-426R2 | CTTTWAGAAGTATCGGGACA |  |
|  |  |  |  |
|  | HTNV-S-245F | CAACMGGRAAGAATCTYGGA | 487 |
|  | HTNV-S-846R1 | CATATTGCCYAATGCCACT |  |
|  | HTNV-S-732R2 | TTGCTCAATGCGGTCACTC |  |
|  |  |  |  |
|  | HTNV-S-585F1 | TGCACAGTCAAGTATGAARGC | 386 |
|  | HTNV-S-665F2 | CCTGCACAAATTAAGGCAAR |  |
|  | HTNV-S-1051R | GATGCCATGATHGTATTCCG |  |
|  |  |  |  |
|  | HTNV-S-996F | TTTCATTGCAGGTATTGCTGA | 427 |
|  | HTNV-S-1489R1 | CAGCCAGGAACTAAACCCA |  |
|  | HTNV-S-1423R2 | CATAAATGKKCCCTTACCCT |  |
|  |  |  |  |
|  | HTNV-S-1230F1 | AGGAGATGATATGGATCC | 423 |
|  | HTNV-S-1259F2 | CATTGGCACAGAGCCTGATT |  |
|  | HTNV-S-1682R | TAGTAGTAGTATGCTCCCTAA |  |
|  |  |  |  |
|  | HTNV-M-17F | AAARGAAARVMRTCAATCAGC | 584 |
|  | HTNV-M-686R1 | GAGTTTATAGGTGTTCCCCTT |  |
|  | HTNV-M-601R2 | CTAACAACACTTTGATCTGGG |  |
|  |  |  |  |
|  | HTNV-M-501F | TGAAGAGCTGTYTAATTGCAT | 590 |
|  | HTNV-M-1118R1 | TAAATCAATCATCCCAGTCCA |  |
|  | HTNV-M-1091R2 | TGGTATTGCACTCTTATCACA |  |
|  |  |  |  |
|  | HTNV-M-1063F | ATTGTTCCCRAARCTTAACCA | 391 |
|  | HTNV-M-1658R1 | ATCACATACCATTGAACCCTT |  |
|  | HTNV-M-1454R2 | TCCAGGCACACATARTTCCAC |  |
|  |  |  |  |
|  | HTNV-M-1259F | GTTTCAAARCAAAATCGGTTC | 617 |
|  | HTNV-M-1935R1 | AATGAGAAGGAATACCCAMW |  |
|  | HTNV-M-1876R2 | ATTTAATGTCCGATAACAMCCT |  |
|  |  |  |  |
|  | HTNV-M-1805F | AAAGTATGCCAGGTTACTCAC | 436 |
|  | HTNV-M-2319R1 | TTACAGCCCCAACTTGTCT |  |
|  | HTNV-M-2241R2 | TGTACAAGCACCATAACAGT |  |
|  |  |  |  |
|  | HTNV-M-2190F | ACGCTYTAGGACATTGGTTT | 644 |
|  | HTNV-M-2870R1 | TCCCTCAACATCCCATCAGG |  |
|  | HTNV-M-2834R2 | CCTCTCATCAGTAAAGTGCAT |  |
|  |  |  |  |
|  | HTNV-M-2790F | TGATGGCAACAATYGACTCT | 434 |
|  | HTNV-M-3307R1 | TCCCCKGAHTTGACAAACCAA |  |
|  | HTNV-M-3224R2 | ATCCCATTAACTTTGTCAAGGTG |  |
|  |  |  |  |
|  | HTNV-M-3151F | TCATAGTGGTTCAACTTTC | 302 |
|  | HTNV-M-3509R1 | GTTAGCTAAAGTGTCCAGGAA |  |
|  | HTNV-M-3453R2 | TAACTTGATAGCGGTATCAT |  |
|  |  |  |  |
|  | HTNV-L-15F | CTAAGTGAMMMACCTYGAA | 722 |
|  | HTNV-L-860R1 | TCTGACAATGATTTGCCTC |  |
|  | HTNV-L-737R2 | TATTTGCTCCTATCGGCTC |  |
|  |  |  |  |
|  | HTNV-L-525F1 | CAGATGGTTCTAATATCACT | 535 |
|  | HTNV-L-723F2 | CVSCHHHATTAYATACCAGA |  |
|  | HTNV-L-1258R | CTGCACAATATGACTAGCT |  |
|  |  |  |  |
|  | HTNV-L-1100F | CCACGAATMCAGTTYAAGCA | 542 |
|  | HTNV-L-1691R1 | TTGATACTCCCCATTGTGA |  |
|  | HTNV-L-1642R2 | RTCCTTATCCACAAGACCG |  |
|  |  |  |  |
|  | HTNV-L-1459F | TATAGGCCATCTAATAAGGGA | 584 |
|  | HTNV-L-2151R1 | TTTATAAACTATACGCGACA |  |
|  | HTNV-L-2043R2 | TTATTCTGYGCAAGAGCAM |  |
|  |  |  |  |
|  | HTNV-L-1758F | TTAGCTTTAAACATAGCCTT | 523 |
|  | HTNV-L-2407R1 | TTCTATTGSTKCTAACTCT |  |
|  | HTNV-L-2281R2 | TTTCTCTGAATTTAAGTGCC |  |
|  |  |  |  |
|  | HTNV-L-2049F | TTGCRCAGAATAATAAGGCHM | 579 |
|  | HTNV-L-2762R1 | ATGGCATCATAATAATCCTC |  |
|  | HTNV-L-2628R2 | YTCATACAACTTTAAGAGGC |  |
|  |  |  |  |
|  | HTNV-L-2519F1 | ATTAGTTTAAAAGGTATGTCCG | 352 |
|  | HTNV-L-2580F2 | CAATTATAGAAGCTATCCGTT |  |
|  | HTNV-L-2932R | TGCACTAACATACATGAGT |  |
|  |  |  |  |
|  | HTNV-L-2709F1 | ATAGAGGCTTCTTTATCACA | 804 |
|  | HTNV-L-2766F2 | ATTATTATGATGCCATTGCTA |  |
|  | HTNV-L-3570R | TGGTATTGWWMCAGCACAAC |  |
|  |  |  |  |
|  | HTNV-L-3396F | TKTYYMAACASAWTCAAGC | 809 |
|  | HTNV-L-4263R1 | CTTCATATGTTGTTTGGCTT |  |
|  | HTNV-L-4205R2 | ATCGCTGTGACTATACTTGG |  |
|  |  |  |  |
|  | HTNV-L-4103F | AAATTTCTWRAATTATGGAGC | 691 |
|  | HTNV-L-4882R1 | GYCCTATATYTTCTTTACGA |  |
|  | HTNV-L-4794R2 | GATTTYAACCAGTATGCAC |  |
|  |  |  |  |
|  | HTNV-L-4562F | TATAAATTTGCTGTAACTGTA | 664 |
|  | HTNV-L-5257R1 | TCCCAGAAGATCTCAAGCCTY |  |
|  | HTNV-L-5226R2 | GACCAAAATTAAATGTGACCC |  |
|  |  |  |  |
|  | HTNV-L-5137F | TAATAGGCGRGCTTACTCA | 514 |
|  | HTNV-L-5789R1 | WCTGATATCTTTGCTCGGTT |  |
|  | HTNV-L-5651R2 | CATACATTACTTACRGCATC |  |
|  |  |  |  |
|  | HTNV-L-5490F | TAAGAACMAGCCTTAGGAC | 624 |
|  | HTNV-L-6350R1 | CTTTGRGCCTCTCTATCAAG |  |
|  | HTNV-L-6114R2 | ATTTTCACTAYRCCCCTCA |  |
|  |  |  |  |
|  | HTNV-L-5827F1 | TGACCTACTCAAGCTAATTA | 530 |
|  | HTNV-L-5984F2 | ACAATTGACTTGCAGGATGT |  |
|  | HTNV-L-6514R | TAGTAGTAGTATGCTCCGGA |  |
|  |  |  |  |
| Amur virus | AMRV-S-14F | CCTAAAGAGCTACTATAAC | 486 |
|  | AMRV-S-532R1 | CGAATKCCRTTYACATCCTC |  |
|  | AMRV-S-500R2 | CATCTTTGAATCGGATYCTTG |  |
|  |  |  |  |
|  | AMRV-S-414F | TACATCCTTTGTRGTTCCGAT | 496 |
|  | AMRV-S-986R1 | CCCTRCAATGAATAAACATGTYG |  |
|  | AMRV-S-910R2 | GTCTTCTATCATGCTACAGCC |  |
|  |  |  |  |
|  | AMRV-S-820F | AATAGAGATTATCTTCGGCAG | 586 |
|  | AMRV-S-1489R1 | GCAGCTTGAAACTAAACCC |  |
|  | AMRV-S-1406R2 | CCTGATATGTTGATTCCCC |  |
|  |  |  |  |
|  | AMRV-M-19F | AAGAAAGCAGTTAATCAGC | 592 |
|  | AMRV-M-635R1 | ACTTGCAATATCAAATATRCC |  |
|  | AMRV-M-611R2 | TTTTATAATRCTCACAACACT |  |
|  |  |  |  |
|  | AMRV-M-502F | GAAGAGCTGYCTAATAGCAT | 587 |
|  | AMRV-M-1132R1 | CTTCATAATAACCAGGTAGRTCA |  |
|  | AMRV-M-1089R2 | GTATGGCAGTTTTATCACACC |  |
|  |  |  |  |
|  | AMRV-M-1033F | TACAGATGCCCGYCATGTC | 634 |
|  | AMRV-M-1714R1 | TGATACTCCATGTGCCTTC |  |
|  | AMRV-M-1667R2 | TTTACACACATCGCAAACCAT |  |
|  |  |  |  |
|  | AMRV-M-1598F | AATCAGGAAAATCGGCTCA | 540 |
|  | AMRV-M-2249R1 | ATTCATATTTTGTACATGCACC |  |
|  | AMRV-M-2138R2 | ACTCAATATGAAGATCTACTGC |  |
|  |  |  |  |
|  | AMRV-M-2095F | CACTTATCGACGGAAGCTC | 510 |
|  | AMRV-M-2750R1 | ATGTTCCCGTCATAYTCRCA |  |
|  | AMRV-M-2605R2 | ACCAATGTTTGAAGATTARGCCA |  |
|  |  |  |  |
|  | AMRV-M-2480F | YTGTGCAAGGTAATTGACAT | 646 |
|  | AMRV-M-3159R1 | ACAGCATTTAAAGGTTGAGC |  |
|  | AMRV-M-3126R2 | TTCCCTGAAACTTTGACRG |  |
|  |  |  |  |
|  | AMRV-M-3004F1 | CTTCACACTGACATGTCAAG | 365 |
|  | AMRV-M-3099F2 | GCATAACACTTACAAGGG |  |
|  | AMRV-M-3464R | TATAAATGAAGTAGTTATGTG |  |
|  |  |  |  |
|  | AMRV-L-11F | CTCCGTAAGTAACAAGAGC | 569 |
|  | AMRV-L-651R1 | TTAAACATTGCCTCTAAAGCAG |  |
|  | AMRV-L-580R2 | CTGCACTAATCTCATATACTGG |  |
|  |  |  |  |
|  | AMRV-L-538F | TATCACAACTCAATGGCCTA | 451 |
|  | AMRV-L-1078R1 | GTGCATAATAATTTATCATGTCT |  |
|  | AMRV-L-989R2 | GAGGTTRAGAATATATGCACA |  |
|  |  |  |  |
|  | AMRV-L-938F | TCATATAAGCCTGCAACCAC | 550 |
|  | AMRV-L-1551R1 | ATAAATAGAATCACATTGCCAT |  |
|  | AMRV-L-1488R2 | AGAATGGGCAATTAAACTCT |  |
|  |  |  |  |
|  | AMRV-L-1093F1 | TTATGCACCAAGGAYYCAG | 592 |
|  | AMRV-L-1140F2 | CWGGCACATTTAAGTTGACA |  |
|  | AMRV-L-1762R | TGTTGCAATTAAMGCCTTC |  |
|  |  |  |  |
|  | AMRV-L-1726F | TTTRAACAGACTACTGGCAT | 401 |
|  | AMRV-L-2153R1 | CTGATCTACAGTTARCCCTA |  |
|  | AMRV-L-2127R2 | TGAAGGATAAATTCCGCTTG |  |
|  |  |  |  |
|  | AMRV-L-2062F | TAAGGCACSGTTTTACTCA | 493 |
|  | AMRV-L-2669R1 | TTTCCTGACTATCCTAGCCAT |  |
|  | AMRV-L-2555R2 | GAGGAAGATAAATGCCCATC |  |
|  |  |  |  |
|  | AMRV-L-2497F | TTACTTCAGYCAAACYAGGAA | 498 |
|  | AMRV-L-3022R1 | CTTTAATTTATCATTRGGGAGC |  |
|  | AMRV-L-2995R2 | TGTAACATGGCAGTAAACCTC |  |
|  |  |  |  |
|  | AMRV-L-2820F | GTGAAAAGAAGATTTTAGCCAT | 750 |
|  | AMRV-L-3602R1 | TCTGATARTGATCCAAGCAG |  |
|  | AMRV-L-3570R2 | TGGAATRGAAACAGCACAAC |  |
|  |  |  |  |
|  | AMRV-L-3526F | TGTTTCCCCTACTAATGCAGA | 468 |
|  | AMRV-L-4020R1 | TRCCAATAAATGARAACTGAC |  |
|  | AMRV-L-3994R2 | YTTYSKTGTTGAAAAGTCTCA |  |
|  |  |  |  |
|  | AMRV-L-3992F | GATGAGACTTTTCAACAMSRA | 560 |
|  | AMRV-L-4668R1 | TATATCAAGCCCAAGTTCCC |  |
|  | AMRV-L-4552R2 | CRAACTTGTASCCTATGACA |  |
|  |  |  |  |
|  | AMRV-L-4106F | TTTCTGGAACTATGGAGCAA | 678 |
|  | AMRV-L-4828R1 | GGGGCTTAACCCTTATWGTT |  |
|  | AMRV-L-4784R2 | CAGTAGGCACAAATAGCTTC |  |
|  |  |  |  |
|  | AMRV-L-4704F | TGAAAAGARTTGCACCCAT | 522 |
|  | AMRV-L-5254R1 | AAAGATCTCYAGCCTAGCA |  |
|  | AMRV-L-5226R2 | GCCCAAAATTAAATGTAACCC |  |
|  |  |  |  |
|  | AMRV-L-4968F | CACCATAYARAGAATGGYT | 637 |
|  | AMRV-L-5763R1 | AATGAGTATGCAGCAGGTG |  |
|  | AMRV-L-5605R2 | TTGTATGAAATGCATGTGCT |  |
|  |  |  |  |
|  | AMRV-L-5490F | TAAGAACMAGCCTTAGGAC | 624 |
|  | AMRV-L-6350R1 | CTTTGRGCCTCTCTATCAAG |  |
|  | AMRV-L-6114R2 | ATTTTCACTAYRCCCCTCA |  |
|  |  |  |  |
|  | AMRV-L-5943F1 | TGAGTTTAGTGGATGATGAT | 499 |
|  | AMRV-L-6016F2 | CATCGACATTGAGGCAGACA |  |
|  | AMRV-L-6515R | TAGTAGTAGTATGCTCCGG |  |

* F: Forward; R: Reverse.
