# Supplementary material for: A molecular survey of orthohantaviruses in rodents across the tri-border region of China, Russia, and North Korea
Source: PLoS Negl Trop Dis. 2026 Apr 20;20(4):e0014134. doi: 10.1371/journal.pntd.0014134 (PMC13120696; doi:10.1371/journal.pntd.0014134)
Supplement: S2 Table — (DOCX) [file pntd.0014134.s009.docx]

**S3 Table.** Reference orthohantavirus strains used for phylogenetic analysis.

| **Segment** | **Accession number** | **Strain** | **Host** | **Country** |
| --- | --- | --- | --- | --- |
| L | AB620030 | Khekhtsir/AP209/2005 | *Apodemus peninsulae* | Russia |
|  | JX473002 | ApJLCB2011-99 | *Apodemus peninsulae* | China |
|  | KC136242 | H8205 | *Homo sapiens* | China |
|  | AB620033 | Galkino/AA57/2002 | *Apodemus agrarius* | Russia |
|  | KJ857317 | Fuyuan-Aa-26 | *Apodemus agrarius* | China |
|  | KY594712 | Aa 08-1111 | *Apodemus agrarius* | South Korea |
|  | KT885047 | 76-118/POR | *Apodemus agrarius* | South Korea |
|  | PQ212937 | Aa22-65 | *Apodemus agrarius* | South Korea |
|  | KX687237 | Aa13_3 | *Apodemus agrarius* | South Korea |
|  | MK548667 | Aa16-50-P | *Apodemus agrarius* | South Korea |
|  | OR113707 | Aa17-1_CU | *Apodemus agrarius* | South Korea |
|  | MW219757 | Ac20-5 | *Apodemus chejuensis* | South Korea |
|  | KX289952 | DN2 | *Rattus norvegicus* | China |
|  | JX853574 | DPRK08 | *Rattus norvegicus* | North Korea |
|  | MT711942 | LN03 | *Rattus norvegicus* | China |
|  | OK500096 | SR-11 | *Rat* | Japan |
|  | MF149937 | Rn10-145 | *Rattus norvegicus* | South Korea |
|  | MF149938 | Rn10-134/NGS | *Rattus norvegicus* | South Korea |
|  | NC034399 | 10-11 | *Crocidura lasiura* | South Korea |
|  | MN432481 | 17-51 | *Crocidura shantungensis* | South Korea |
|  | KJ857320 | Fuyuan-Sr-326 | *Sorex roboratus* | China |
|  | MH499473 | Galkino-St2714/Russia/2007 | *Sorex tundrensis* | Russia |
|  | KJ857311 | Fuyuan-Mm-250 | *Microtus maximowiczii* | China |
|  | JX028271 | 11-1 | *Myodes regulus* | South Korea |
|  | MZ014471 | Saratov-MG120/Russia/2019 | *Myodes glareolus* | Russia |
| M | AB620029 | Khekhtsir/AP209/2005 | *Apodemus peninsulae* | Russia |
|  | JX473003 | ApJLCB2011-99 | *Apodemus peninsulae* | China |
|  | JX119009 | JLCB2011 | *Apodemus peninsulae* | China |
|  | AB127993 | H5 | *Homo sapiens* | China |
|  | EF371454 | JilinAP06 | *Apodemus peninsulae* | China |
|  | AB127994 | B78 | *Homo sapiens* | China |
|  | KC136243 | H8205 | *Homo sapiens* | China |
|  | KM853165 | NMAp117 | *Apodemus peninsulae* | China |
|  | KC576786 | CBAa11-26 | *Apodemus agrarius* | China |
|  | AB620032 | Galkino/AA57/2002 | *Apodemus agrarius* | Russia |
|  | KJ857334 | Fuyuan-Aa-26 | *Apodemus agrarius* | China |
|  | AY748307 | CJilin93 | *Apodemus peninsulae* | China |
|  | EF208930 | CJAp93 | *Apodemus peninsulae* | China |
|  | AB127995 | Bao14 | *Apodemus agrarius* | China |
|  | OR711250 | HNTV/Apodemus_agrarius/Russia (Primorye)/FE-5454/2010 | *Apodemus agrarius* | Russia |
|  | KT885048 | 76-118/POR | *Apodemus agrarius* | South Korea |
|  | KY594715 | Aa 08-1111 | *Apodemus agrarius* | South Korea |
|  | PQ247660 | Aa22-65 | *Apodemus agrarius* | South Korea |
|  | KX687227 | Aa13_3 | *Apodemus agrarius* | South Korea |
|  | MK548658 | Aa16-50-P | *Apodemus agrarius* | South Korea |
|  | OR113716 | Aa17-1_CU | *Apodemus agrarius* | South Korea |
|  | MW219763 | Ac20-5 | *Apodemus chejuensis* | South Korea |
|  | KX289953 | DN2 | *Rattus norvegicus* | China |
|  | OK500097 | SR-11 | *Rat* | Japan |
|  | MF149942 | Rn10-134/NGS | *Rattus norvegicus* | South Korea |
|  | GU592923 | HuludaoRn101 | *Rattus norvegicus* | China |
|  | JX853576 | DPRK08 | *Rattus norvegicus* | North Korea |
|  | MT711948 | LN03 | *Rattus norvegicus* | China |
|  | NC034404 | 10-11 | *Crocidura lasiura* | South Korea |
|  | EU929075 | N10 | *Urotrichus talpoides* | Japan |
|  | KJ857337 | Fuyuan-Sr-326 | *Sorex roboratus* | China |
|  | MG913806 | Galkino-St2714/Russia/2007 | *Sorex tundrensis* | Russia |
|  | EU072488 | Fusong-Mf-682 | *Microtus fortis* | China |
|  | KJ857340 | Fuyuan-Mm-250 | *Microtus maximowiczii* | China |
|  | JX028272 | 11-1 | *Myodes regulus* | South Korea |
|  | MZ014468 | Saratov-MG120/Russia/2019 | *Myodes glareolus* | Russia |
| S | AB620028 | Khekhtsir/AP209/2005 | *Apodemus peninsulae* | Russia |
|  | JX473004 | ApJLCB2011-99 | *Apodemus peninsulae* | China |
|  | JX119010 | JLCB2011 | *Apodemus peninsulae* | China |
|  | JQ061291 | NA33 | *Apodemus peninsulae* | China |
|  | EF121324 | JilinAP06 | *Apodemus peninsulae* | China |
|  | KC136244 | H8205 | *Homo sapiens* | China |
|  | AB127996 | H5 | *Homo sapiens* | China |
|  | AB127997 | B78 | *Homo sapiens* | China |
|  | KM853162 | NMAp117 | *Apodemus peninsulae* | China |
|  | KM355414 | WCL | *Homo sapiens* | China |
|  | AB127998 | Bao14 | *Apodemus agrarius* | China |
|  | AB620031 | Galkino/AA57/2002 | *Apodemus agrarius* | Russia |
|  | KJ857347 | Fuyuan-Aa-26 | *Apodemus agrarius* | China |
|  | HQ611981 | YaluRiver13 | *Apodemus agrarius* | China |
|  | EF208929 | CJAp93 | *Apodemus peninsulae* | China |
|  | OR711249 | HNTV/Apodemus_agrarius/Russia (Primorye)/FE-5454/2010 | *Apodemus agrarius* | Russia |
|  | KX687232 | Aa13_3 | *Apodemus agrarius* | South Korea |
|  | MK548649 | Aa16-50-P | *Apodemus agrarius* | South Korea |
|  | PQ247675 | Aa22-65 | *Apodemus agrarius* | South Korea |
|  | KT885049 | 76-118/POR | *Apodemus agrarius* | South Korea |
|  | KY594718 | Aa 08-1111 | *Apodemus agrarius* | South Korea |
|  | MW219769 | Ac20-5 | *Apodemus chejuensis* | South Korea |
|  | OR113725 | Aa17-1_CU | *Apodemus agrarius* | South Korea |
|  | GU592952 | HuludaoRn101 | *Rattus norvegicus* | China |
|  | HQ611980 | YaluRiver12 | *Rattus norvegicus* | North Korea |
|  | JX853575 | DPRK08 | *Rattus norvegicus* | North Korea |
|  | MT711954 | LN03 | *Rattus norvegicus* | China |
|  | KX289954 | DN2 | *Rattus norvegicus* | China |
|  | OK500098 | SR-11 | *Rat* | Japan |
|  | NC005236 | 80-39 | *Rattus norvegicus* | South Korea |
|  | MF149947 | Rn10-134/NGS | *Rattus norvegicus* | South Korea |
|  | EU072480 | Fusong-Mf-682 | *Microtus fortis* | China |
|  | KJ857344 | Fuyuan-Mm-250 | *Microtus maximowiczii* | China |
|  | JX028273 | 11-1 | *Myodes regulus* | South Korea |
|  | MZ014465 | Saratov-MG120/Russia/2019 | *Myodes glareolus* | Russia |
|  | NC034398 | 10-11 | *Crocidura lasiura* | South Korea |
|  | EU929072 | N10 | *Urotrichus talpoides* | Japan |
|  | KJ857341 | Fuyuan-Sr-326 | *Sorex roboratus* | China |
|  | MG888402 | Galkino-St2714/Russia/2007 | *Sorex tundrensis* | Russia |

The sequence highlighted in light green indicates a discrepancy between the data recorded in the NCBI database and the information reported in the original publication. For this study, the data from the original publication were prioritized and used as the reference.
