# Supplementary material for: A molecular survey of orthohantaviruses in rodents across the tri-border region of China, Russia, and North Korea
Source: PLoS Negl Trop Dis. 2026 Apr 20;20(4):e0014134. doi: 10.1371/journal.pntd.0014134 (PMC13120696; doi:10.1371/journal.pntd.0014134)
Supplement: S3 Table — (DOCX) [file pntd.0014134.s010.docx]

**S4 Table.** Reference orthohantavirus strains used for homology analysis.

| **Segment** | **Accession number** | **Strain** | **Host** | **Country** |
| --- | --- | --- | --- | --- |
| L | AB620030 | Khekhtsir/AP209/2005 | *Apodemus peninsulae* | Russia |
|  | JX473002 | ApJLCB2011-99 | *Apodemus peninsulae* | China |
|  | KC136242 | H8205 | *Homo sapiens* | China |
|  | KJ857317 | Fuyuan-Aa-26 | *Apodemus agrarius* | China |
|  | AB620033 | Galkino/AA57/2002 | *Apodemus agrarius* | Russia |
|  | KT885047 | 76-118/POR | *Apodemus agrarius* | South Korea |
|  | PQ212937 | Aa22-65 | *Apodemus agrarius* | South Korea |
|  | KX687237 | Aa13_3 | *Apodemus agrarius* | South Korea |
|  | MK548667 | Aa16-50-P | *Apodemus agrarius* | South Korea |
|  | KY594712 | Aa 08-1111 | *Apodemus agrarius* | South Korea |
|  | JX853574 | DPRK08 | *Rattus norvegicus* | North Korea |
|  | MT711942 | LN03 | *Rattus norvegicus* | China |
|  | OK500096 | SR-11 | *Rat* | Japan |
|  | MF149938 | Rn10-134/NGS | *Rattus norvegicus* | South Korea |
|  | KX289952 | DN2 | *Rattus norvegicus* | China |
|  | KJ857320 | Fuyuan-Sr-326 | *Sorex roboratus* | China |
|  | MH499473 | Galkino-St2714/Russia/2007 | *Sorex tundrensis* | Russia |
|  | NC034399 | 10-11 | *Crocidura lasiura* | South Korea |
|  | JX028271 | 11-1 | *Myodes regulus* | South Korea |
|  | MZ014471 | Saratov-MG120/Russia/2019 | *Myodes glareolus* | Russia |
|  | KJ857311 | Fuyuan-Mm-250 | *Microtus maximowiczii* | China |
| M | AB620029 | Khekhtsir/AP209/2005 | *Apodemus peninsulae* | Russia |
|  | JX473003 | ApJLCB2011-99 | *Apodemus peninsulae* | China |
|  | KC136243 | H8205 | *Homo sapiens* | China |
|  | KJ857334 | Fuyuan-Aa-26 | *Apodemus agrarius* | China |
|  | AB620032 | Galkino/AA57/2002 | *Apodemus agrarius* | Russia |
|  | KT885048 | 76-118/POR | *Apodemus agrarius* | South Korea |
|  | PQ247660 | Aa22-65 | *Apodemus agrarius* | South Korea |
|  | KX687227 | Aa13_3 | *Apodemus agrarius* | South Korea |
|  | MK548658 | Aa16-50-P | *Apodemus agrarius* | South Korea |
|  | KY594715 | Aa 08-1111 | *Apodemus agrarius* | South Korea |
|  | JX853576 | DPRK08 | *Rattus norvegicus* | North Korea |
|  | MT711948 | LN03 | *Rattus norvegicus* | China |
|  | OK500097 | SR-11 | *Rat* | Japan |
|  | MF149942 | Rn10-134/NGS | *Rattus norvegicus* | South Korea |
|  | KX289953 | DN2 | *Rattus norvegicus* | China |
|  | KJ857337 | Fuyuan-Sr-326 | *Sorex roboratus* | China |
|  | MG913806 | Galkino-St2714/Russia/2007 | *Sorex tundrensis* | Russia |
|  | NC034404 | 10-11 | *Crocidura lasiura* | South Korea |
|  | JX028272 | 11-1 | *Myodes regulus* | South Korea |
|  | MZ014468 | Saratov-MG120/Russia/2019 | *Myodes glareolus* | Russia |
|  | KJ857340 | Fuyuan-Mm-250 | *Microtus maximowiczii* | China |
| S | AB620028 | Khekhtsir/AP209/2005 | *Apodemus peninsulae* | Russia |
|  | JX473004 | ApJLCB2011-99 | *Apodemus peninsulae* | China |
|  | KC136244 | H8205 | *Homo sapiens* | China |
|  | KJ857347 | Fuyuan-Aa-26 | *Apodemus agrarius* | China |
|  | AB620031 | Galkino/AA57/2002 | *Apodemus agrarius* | Russia |
|  | KT885049 | 76-118/POR | *Apodemus agrarius* | South Korea |
|  | PQ247675 | Aa22-65 | *Apodemus agrarius* | South Korea |
|  | KX687232 | Aa13_3 | *Apodemus agrarius* | South Korea |
|  | MK548649 | Aa16-50-P | *Apodemus agrarius* | South Korea |
|  | KY594718 | Aa 08-1111 | *Apodemus agrarius* | South Korea |
|  | JX853575 | DPRK08 | *Rattus norvegicus* | North Korea |
|  | MT711954 | LN03 | *Rattus norvegicus* | China |
|  | OK500098 | SR-11 | *Rat* | Japan |
|  | MF149947 | Rn10-134/NGS | *Rattus norvegicus* | South Korea |
|  | KX289954 | DN2 | *Rattus norvegicus* | China |
|  | KJ857341 | Fuyuan-Sr-326 | *Sorex roboratus* | China |
|  | MG888402 | Galkino-St2714/Russia/2007 | *Sorex tundrensis* | Russia |
|  | NC034398 | 10-11 | *Crocidura lasiura* | South Korea |
|  | JX028273 | 11-1 | *Myodes regulus* | South Korea |
|  | MZ014465 | Saratov-MG120/Russia/2019 | *Myodes glareolus* | Russia |
|  | KJ857344 | Fuyuan-Mm-250 | *Microtus maximowiczii* | China |

The sequence highlighted in light green indicates a discrepancy between the data recorded in the NCBI database and the information reported in the original publication. For this study, the data from the original publication were prioritized and used as the reference.
