# Supplementary material for: A molecular survey of orthohantaviruses in rodents across the tri-border region of China, Russia, and North Korea
Source: PLoS Negl Trop Dis. 2026 Apr 20;20(4):e0014134. doi: 10.1371/journal.pntd.0014134 (PMC13120696; doi:10.1371/journal.pntd.0014134)
Supplement: S4 Table — (DOCX) [file pntd.0014134.s011.docx]

**S5 Table.** Statistical analyses of the prevalence of Hantaan virus and Amur virus in rodents collected from the tri-border region of China, Russia, and North Korea.

| Variables | Categories | No. positive/Total  (n/N) | Prevalence (%) | χ² | *p*-Value |
| --- | --- | --- | --- | --- | --- |
| Year | 2022 (HTNV, *A. agrarius*) | 8/92 | 8.7 | 0.018 | 0.894 |
|  | 2023 (HTNV, *A. agrarius*) | 7/86 | 8.1 |  |  |
|  |  |  |  |  |  |
|  | 2022 (HTNV, *R. norvegicus*) | 1/52 | 1.9 | – | 0.621^a^ |
|  | 2023 (HTNV, *R. norvegicus*) | 3/59 | 5.1 |  |  |
|  |  |  |  |  |  |
|  | 2022 (AMRV, *A. peninsulae*) | 2/33 | 6.1 | – | 1.000^a^ |
|  | 2023 (AMRV, *A. peninsulae*) | 3/46 | 6.5 |  |  |
|  |  |  |  |  |  |
| Season | Spring (HTNV, *A. agrarius*) | 6/67 | 9.0 | 0.039 | 0.844 |
|  | Autumn (HTNV, *A. agrarius*) | 9/111 | 8.1 |  |  |
|  |  |  |  |  |  |
|  | Spring (HTNV, *R. norvegicus*) | 3/73 | 4.1 | – | 1.000^a^ |
|  | Autumn (HTNV, *R. norvegicus*) | 1/38 | 2.6 |  |  |
|  |  |  |  |  |  |
|  | Spring (AMRV, *A. peninsulae*) | 1/22 | 4.5 | – | 1.000^a^ |
|  | Autumn (AMRV, *A. peninsulae*) | 4/57 | 7.0 |  |  |
|  |  |  |  |  |  |
| Area | Residential area (HTNV, *A. agrarius*) | 1/9 | 11.1 | – | 0.566^a^ |
|  | Farmland (HTNV, *A. agrarius*) | 9/116 | 7.8 |  |  |
|  | Wetland (HTNV, *A. agrarius*) | 2/33 | 6.1 |  |  |
|  | Forest (HTNV, *A. agrarius*) | 3/20 | 15.0 |  |  |
|  |  |  |  |  |  |
|  | Residential area (HTNV, *R. norvegicus*) | 3/96 | 3.1 | – | 0.278^a^ |
|  | Farmland (HTNV, *R. norvegicus*) | 1/5 | 20.0 |  |  |
|  | Wetland (HTNV, *R. norvegicus*) | 0/7 | 0 |  |  |
|  | Forest (HTNV, *R. norvegicus*) | 0/3 | 0 |  |  |
|  |  |  |  |  |  |
|  | Residential area (AMRV, *A. peninsulae*) | 0/0 | 0 | N/A | N/A |
|  | Farmland (AMRV, *A. peninsulae*) | 0/0 | 0 |  |  |
|  | Wetland (AMRV, *A. peninsulae*) | 0/0 | 0 |  |  |
|  | Forest (AMRV, *A. peninsulae*) | 5/79 | 6.3 |  |  |
|  |  |  |  |  |  |
| Rodent species | *R. norvegicus* (HTNV) | 4/111 | 3.6 | 2.590 | 0.108 |
|  | *A. agrarius* (HTNV) | 15/178 | 8.4 |  |  |

^a^ p values were calculated using Fisher's exact test due to small sample sizes (expected count < 5). N/A, not applicable. statistical comparisons were precluded by the lack of sufficient samples in these categories.
