# Supplementary material for: A molecular survey of orthohantaviruses in rodents across the tri-border region of China, Russia, and North Korea
Source: PLoS Negl Trop Dis. 2026 Apr 20;20(4):e0014134. doi: 10.1371/journal.pntd.0014134 (PMC13120696; doi:10.1371/journal.pntd.0014134)
Supplement: S5 Table — (DOCX) [file pntd.0014134.s012.docx]

S6 Table. Statistical analyses of viral titers among different host and virus species.

| Categories | N | Median (IQR) | Mann-Whitney U | *Z* Score | *P*-Value |
| --- | --- | --- | --- | --- | --- |
| HTNV (*R. norvegicus*) | 4 | 5.62 (4.33–6.74) | 23 | -0.70 | 0.484 |
| HTNV (*A. agrarius*) | 15 | 5.38 (3.88–6.09) |  |  |  |
|  |  |  |  |  |  |
| HTNV (*A. agrarius*) | 15 | 5.38 (3.88–6.09) | 29 | -0.742 | 0.458 |
| AMRV (*A. peninsulae*) | 5 | 6.17 (3.80–6.47) |  |  |  |
|  |  |  |  |  |  |
| HTNV (*R. norvegicus)* | 4 | 5.62 (4.33–6.74) | 7 | -0.735 | 0.462 |
| AMRV (*A. peninsulae*) | 5 | 6.17 (3.80–6.47) |  |  |  |

Data are presented as median (interquartile range, IQR)
