# Supplementary material for: A molecular survey of orthohantaviruses in rodents across the tri-border region of China, Russia, and North Korea
Source: PLoS Negl Trop Dis. 2026 Apr 20;20(4):e0014134. doi: 10.1371/journal.pntd.0014134 (PMC13120696; doi:10.1371/journal.pntd.0014134)
Supplement: S6 Table — (DOCX) [file pntd.0014134.s013.docx]

**S7 Table.** Information on the 11 complete genome sequences of the amplified viral strains in this study.

| **Strain** | **Accession number** | **Virus** | **Collection sites** | **Host** |
| --- | --- | --- | --- | --- |
| Yanbian-Aa22s-H3981 | PV600322 (L) | *Hantaan virus* | Hunchun, China | *Apodemus agrarius* |
|  | PV600333 (M) |  |  |  |
|  | PV600344 (S) |  |  |  |
|  |  |  |  |  |
| Yanbian-Aa22a-H3999 | PV600323 (L) | *Hantaan virus* | Hunchun, China | *Apodemus agrarius* |
|  | PV600334 (M) |  |  |  |
|  | PV600345 (S) |  |  |  |
|  |  |  |  |  |
| Yanbian-Aa22a-H4010 | PV600324 (L) | *Hantaan virus* | Hunchun, China | *Apodemus agrarius* |
|  | PV600335 (M) |  |  |  |
|  | PV600346 (S) |  |  |  |
|  |  |  |  |  |
| Yanbian-Aa22s-H3931 | PV600325 (L) | *Hantaan virus* | Hunchun, China | *Apodemus agrarius* |
|  | PV600336 (M) |  |  |  |
|  | PV600347 (S) |  |  |  |
|  |  |  |  |  |
| Yanbian-Rn22a-H4086 | PV600326 (L) | *Hantaan virus* | Hunchun, China | *Rattus norvegicus* |
|  | PV600337 (M) |  |  |  |
|  | PV600348 (S) |  |  |  |
|  |  |  |  |  |
| Yanbian-Rn22a-H4103 | PV600327 (L) | *Hantaan virus* | Hunchun, China | *Rattus norvegicus* |
|  | PV600338 (M) |  |  |  |
|  | PV600349 (S) |  |  |  |
|  |  |  |  |  |
| Yanbian-Rn22a-H3954 | PV600328 (L) | *Hantaan virus* | Hunchun, China | *Rattus norvegicus* |
|  | PV600339 (M) |  |  |  |
|  | PV600350 (S) |  |  |  |
|  |  |  |  |  |
| Yanbian-Aa22s-H3977 | PV600329 (L) | *Hantaan virus* | Hunchun, China | *Apodemus agrarius* |
|  | PV600340 (M) |  |  |  |
|  | PV600351 (S) |  |  |  |
|  |  |  |  |  |
| Yanbian-Aa22s-H3928 | PV600330 (L) | *Hantaan virus* | Hunchun, China | *Apodemus agrarius* |
|  | PV600341 (M) |  |  |  |
|  | PV600352 (S) |  |  |  |
|  |  |  |  |  |
| Yanbian-Aa23s-H4239 | PV600332 (L) | *Hantaan virus* | Hunchun, China | *Apodemus agrarius* |
|  | PV600343 (M) |  |  |  |
|  | PV600354 (S) |  |  |  |
|  |  |  |  |  |
| Yanbian-Aa22a-H4029 | PV600331 (L) | *Amur virus* | Hunchun, China | *Apodemus peninsulae* |
|  | PV600342 (M) |  |  |  |
|  | PV600353 (S) |  |  |  |
