# Supplementary material for: A molecular survey of orthohantaviruses in rodents across the tri-border region of China, Russia, and North Korea
Source: PLoS Negl Trop Dis. 2026 Apr 20;20(4):e0014134. doi: 10.1371/journal.pntd.0014134 (PMC13120696; doi:10.1371/journal.pntd.0014134)
Supplement: S7 Table — (DOCX) [file pntd.0014134.s014.docx]

**S8 Table.** Information on the remaining viral strains for which partial sequences were amplified in this study.

| **Strain** | **Host** | **Virus** | **Segment** | **Fragment length (bp)** | **Genomic position (nt)** | **Nucleotide identity to our**  **complete genome (%)** | **Sequence (5' → 3')** |
| --- | --- | --- | --- | --- | --- | --- | --- |
| Yanbian-Aa-22a-H4116 | *Apodemus agrarius* | Hantaan virus | L | 1566 | 775–2341 | 99.36–99.94 | CTGTCCAGAGCTAGAAATTTCTCTTTCTTTGAAGTAAAGGGTACAGCAGTTTTCGATTGCTTTAATTCAAATGAGGCAAATCATTGTCAGAAATATCCCATGTCCCGTAAACCTAGGAACTTTCTGTTAATACAATGTTCCTTAATTACATCTTACAAGCCTGCAACTACTCTTTCAGATCAGATTGATAGCAGAAGGGCTTGTTCGTACATCTTGAATCTAATTCCAGACACACCAGCATCATATTTGGTTCATGACATGGCATATAGATATATAAATCTGACAAGAGAAGATATGATTAATTATTATGCCCCACGAATCCAGTTCAAGCAGACACAGAATGTAAGAGAGCCAGGGACATTCAAACTGACATCAAGTATGCTCAGGGCAGAATCAAAAGCAATGCTAGATCTACTTAATAATCATAAAAGTGGTGAGAAGCACGGGGCACAAATAGAAAGTCTAAATATAGCTAGTCATATTGTGCAGTCAGAGTCTGTTAGTTTAATTACAAAGATATTATCTGATCTAGAATTAAACATAACTGAGCCATCAACTCAAGAATATTCTACAACTAAGCACACTTATGTTGACACAGTGCTGGATAAGTTTTTCCAGAATGAGACACAGAAGTATTTAATAGATGTATTGAAGAAGACAACAGCATGGCATATAGGCCATCTAATAAGGGACATAACAGAAAGCTTGATTGCACATTCAGGATTGAAAAGATCAAAGTATTGGTCCCTACATTCTTATAATAATGGAAATGTTATATTATTTATCCTTCCATCAAAATCACTAGAAGTGGCAGGGTCCTTTATTAGATTTATTACAGTTTTTAGGATAGGACCCGGTCTTGTGGATAAGGACAACCTGGATACTATCTTGATTGATGGTGACTCACAATGGGGAGTATCAAAAGTCATGAGTATTGATTTAAACAGGTTGTTAGCTTTAAACATAGCCTTTGAAAAGGCTTTAATTGCAACAGCCACATGGTTTCAATACTACACAGAAGATCAAGGGCAATTTCCCTTGCAATATGCAATTAGATCTGTCTTTGCAAATCATTTCCTATTAGCTATATGTCAGAAGATGAAACTCTGTGCGATTTTTGATAATTTACGCTATTTAATACCTGCAGTAACATCATTGTATTCTGGTTTTCCTTCATTGATAGAAAAGCTCTTCGAACGGCCCTTTAAGTCTTCATTGGAGGTATATATATATTACAATATAAAAAGCTTATTGGTTGCTCTTGCACAGAATAATAAGGCTAGATTCTATTCTAAGGTAAAGTTGTTGGGTTTGACAGTTGACCAATCAACTGTAGGTGCAAGTGGAGTATATCCTTCTTTCATGTCGCGTATAGTTTATAAACATTACAGAAGTTTGATTTCTGAAGTGACAACCTGTTTCTTCTTATTTGAAAAGGGTCTTCATGGTAATATGAATGAAGAAGCAAAGATTCATCTTGAGACAGTAGAATGGGCACTTAAATTCAGAGAAAAAGAGGAAAAGTATGGAGAATCATTGGTGGAAAATGGATATATGATGTGGGAGC |
| Yanbian-Aa22s-H3974 | *Apodemus agrarius* | Hantaan virus | L | 1503 | 776–2279 | 98.94–99.87 | TGTCCAGAGCTAGAAATTTCTCTTTCTTTGAAGTAAAGGGTACAGCAGTTTTCGATTGCTTTAACTCAAATGAGGCAAATCATTGTCAGAAATATCCCATGTCCCGTAAACCTAGGAACTTTCTGTTAATACAATGTTCCTTAATTACATCTTACAAGCCTGCAACTACTCTTTCAGATCAGATTGATAGCAGAAGGGCTTGTTCATACATCTTGAATCTAATTCCAGACACACCAGCATCATATTTGGTTCATGACATGGCATATAGATATATAAATCTGACAAGAGAAGATATGATTAATTATTATGCCCCACGAATCCAGTTCAAGCAGACACAGAATGTAAGAGAGCCAGGGACATTCAAACTGACATCAAGTATGCTCAGGGCAGAATCAAAAGCAATGCTAGATCTACTTAATAATCATAAAAGTGGTGAAAAGCACGGGGCACAAATAGAAAGTCTAAATATAGCTAGTCATATTGTGCAGTCAGAGTCTGTTAGTTTAATTACAAAGATATTATCTGATCTAGAACTAAACATAACTGAGCCATCAACTCAAGAATATTCTACAACTAAGCACACTTATGTTGATACAGTGCTGGATAAGTTCTTTCAGAATGAGACACAGAAGTATTTAATAGATGTATTGAAGAAGACAACAGCATGGCATATAGGCCATCTAATAAGGGACATAACAGAAAGCTTGATTGCACATTCAGGATTGAAAAGATCAAAGTATTGGTCCCTACATTCTTATAATAATGGAAATGTTATATTATTTATCCTTCCATCAAAATCACTAGAAGTGGCAGGGTCCTTTATTAGATTTATTACAGTCTTTAGGATAGGACCCGGTCTTGTGGATAAGGACAACCTGGATACTATCTTGATTGATGGTGACTCACAATGGGGAGTATCAAAAGTCATGAGTATTGATTTAAACAGGTTGTTAGCTTTAAACATAGCCTTTGAAAAGGCTTTAATTGCAACAGCCACATGGTTTCAATACTACACAGAAGATCAAGGGCAATTTCCCTTGCAATATGCAATTAGATCTGTCTTTGCAAATCATTTCCTATTAGCTATATGTCAGAAGATGAAACTCTGTGCGATTTTTGATAATTTGCGCTATTTAATACCTGCAGTAACATCATTGTATTCTGGTTTTCCTTCATTGATAGAAAAGCTCTTTGAACGGCCCTTTAAGTCTTCATTGGAGGTATATATATATTACAATATAAAAAGCTTATTGGTTGCTCTTGCACAGAATAATAAGGCCAGATTCTATTCTAAGGTAAAGTTGTTGGGTTTGACAGTTGACCAATCAACTGTAGGTGCAAGTGGGGTATATCCTTCTTTCATGTCGCGTATAGTTTATAAACATTACAGAAGTTTGATTTCTGAAGTGACAACCTGTTTCTTCTTATTTGAAAAGGGTCTTCATGGTAATATGAATGAAGAAGCAAAGATTCATCTTGAGACAGTAGAATGGGCACTTAAATT |
| Yanbian-Aa22s-H3976 | *Apodemus agrarius* | Hantaan virus | L | 1566 | 775–2341 | 99.30–99.87 | CTGTCCAGAGCTAGAAATTTCTCTTTCTTTGAAGTAAAGGGTACAGCAGTTTTCGATTGCTTTAATTCAAATGAGGCAAATCATTGTCAGAAATATCCCATGTCCCGTAAACCTAGGAACTTTCTGTTAATACAATGTTCCTTAATTACATCTTACAAGCCTGCAACTACTCTTTCAGATCAGATTGATAGCAGAAGGGCTTGTTCGTACATCTTGAATCTAATTCCAGACACACCAGCATCATATTTGGTTCATGACATGGCATATAGATATATAAATCTGACAAGAGAAGATATGATTAATTATTATGCCCCACGAATCCAGTTCAAGCAGACACAGAATGTAAGAGAGCCAGGGACATTCAAACTGACATCAAGTATGCTCAGGGCAGAATCAAAAGCAATGCTAGATCTACTCAATAATCATAAAAGTGGTGAGAAGCACGGGGCACAAATAGAAAGTCTAAATATAGCTAGTCATATTGTGCAGTCAGAGTCTGTTAGTTTAATTACAAAGATATTATCTGATCTAGAATTAAACATAACTGAGCCATCAACTCAAGAATATTCTACAACTAAGCACACTTATGTTGACACAGTGCTGGATAAGTTTTTCCAGAATGAGACACAGAAGTATTTAATAGATGTATTGAAGAAGACAACAGCATGGCATATAGGCCATCTAATAAGGGACATAACAGAAAGCTTGATTGCACATTCAGGATTGAAAAGATCAAAGTATTGGTCCCTACATTCTTATAATAATGGAAATGTTATATTATTTATCCTTCCATCAAAATCACTAGAAGTGGCAGGGTCCTTTATTAGATTTATTACAGTTTTTAGGATAGGACCCGGTCTTGTGGATAAGGACAACCTGGATACTATCTTGATTGATGGTGACTCACAATGGGGAGTATCAAAAGTCATGAGTATTGATTTAAACAGGTTGTTAGCTTTAAACATAGCCTTTGAAAAGGCTTTAATTGCAACAGCCACATGGTTTCAATACTACACAGAAGATCAAGGGCAATTTCCCTTGCAATATGCAATTAGATCTGTCTTTGCAAATCATTTCCTATTAGCTATATGTCAGAAGATGAAACTCTGTGCGATTTTTGATAATTTACGCTATTTAATACCTGCAGTAACATCATTGTATTCTGGTTTTCCTTCATTGATAGAAAAGCTCTTCGAACGGCCCTTTAAGTCTTCATTGGAGGTATATATATATTACAATATAAAAAGCTTATTGGTTGCTCTTGCACAGAATAATAAGGCTAGATTCTATTCTAAGGTAAAGTTGTTGGGTTTGACAGTTGACCAATCAACTGTAGGTGCAAGTGGAGTATATCCTTCTTTCATGTCGCGTATAGTTTATAAACATTACAGAAGTTTGATTTCTGAAGTGACAACCTGTTTCTTCTTATTTGAAAAGGGTCTTCATGGTAATATGAATGAAGAAGCAAAGATTCATCTTGAGACAGTAGAATGGGCACTTAAATTCAGAGAAAAAGAGGAAAAGTATGGAGAATCATTGGTGGAAAATGGATATATGATGTGGGAGC |
| Yanbian-Aa22s-H3979 | *Apodemus agrarius* | Hantaan virus | L | 1594 | 760–2354 | 99.06–99.87 | TATTCTAAAGAATGGCTGTCCAGAGCTAGAAATTTCTCTTTCTTTGAAGTAAAGGGTACAGCAGTTTTCGATTGCTTTAATTCAAATGAGGCAAATCATTGTCAGAAATATCCCATGTCCCGTAAACCTAGGAACTTTCTGTTAATACAATGTTCCTTAATTACATCTTACAAGCCTGCAACTACTCTTTCAGATCAGATTGATAGCAGAAGGGCTTGTTCGTACATCTTGAATCTAATTCCAGACACACCAGCATCATATTTGGTTCATGACATGGCATATAGATATATAAATCTGACAAGAGAAGATATGATTAATTATTATGCCCCACGAATCCAGTTCAAGCAGACACAGAATGTAAGAGAGCCAGGGACATTCAAACTGACATCAAGTATGCTCAGGGCAGAATCAAAAGCAATGCTAGATCTACTTAATAATCATAAAAGTGGTGAGAAGCACGGGGCACAAATAGAAAGTCTAAATATAGCTAGTCATATTGTGCAGTCAGAGTCTGTTAGTTTAATTACAAAGATATTATCTGATCTAGAATTAAACATAACTGAGCCATCAACTCAAGAATATTCTACAACTAAGCACACTTATGTTGATACAATGCTGGATAAGTTCTTCCAGAATGAGACACAGAAGTATTTAATAGATGTATTGAAGAAGACAACAGCATGGCATATAGGCCATCTAATAAGGGACATAACAGAAAGCTTGATTGCACATTCAGGATTGAAAAGATCAAAGTATTGGTCCCTACATTCTTATAATAATGGAAATGTTATATTATTTATCCTTCCATCAAAATCACTAGAAGTGGCAGGGTCCTTTATTAGATTTATTACAGTTTTTAGGATAGGACCCGGTCTTGTGGATAAGGACAACCTGGATACTATCTTGATTGATGGTGACTCACAATGGGGAGTATCAAAAGTCATGAGTATTGATTTAAACAGGTTGTTAGCTTTAAACATAGCCTTTGAAAAGGCTTTAATTGCAACAGCCACATGGTTTCAATACTACACAGAAGATCAAGGGCAATTTCCCTTGCAATATGCAATTAGATCTGTCTTTGCAAATCATTTCCTATTAGCTATATGTCAGAAGATGAAACTCTGTGCGATTTTTGATAATTTGCGCTATTTAATACCTGCAGTAACATCATTGTATTCTGGTTTTCCTTCATTGATAGAAAAGCTCTTCGAACGGCCCTTTAAGTCTTCATTGGAGGTATATATATATTACAATATAAAAAGCTTATTGGTTGCTCTTGCGCAGAATAATAAGGCTAGATTCTATTCTAAGGTAAAGTTGTTGGGTTTGACAGTTGACCAATCAACTGTAGGTGCAAGTGGAGTATATCCTTCTTTCATGTCGCGTATAGTTTATAAACATTACAGAAGTTTGATTTCTGAAGTGACAACCTGTTTCTTCTTATTTGAAAAGGGTCTTCATGGTAATATGAATGAAGAAGCAAAGATTCATCTTGAGACAGTAGAATGGGCACTTAAATTCAGAGAAAAAGAGGAAAAGTATGGAGAATCACTGGTGGAAAATGGATATATGATGTGGGAGCTCAGAGAAAATTC |
| Yanbian-Rn22s-H3873 | *Rattus norvegicus* | Hantaan virus | L | 1610 | 762–2372 | 99.13–99.81 | TTCTAAAGAATGGCTGTCCAGAGCTAGAAATTTCTCTTTCTTTGAAGTAAAGGGTACAGCAGTTTTCGATTGCTTTAATTCAAATGAGGCAAATCATTGTCAGAAATATCCCATGTCCCGTAAACCTAGGAACTTTCTGTTAATACAATGTTCCTTAATTACATCTTACAAGCCTGCAACTACTCTTTCAGATCAGATTGATAGCAGAAGGGCTTGTTCGTACATCTTGAATCTAATTCCAGACACACCAGCATCATATTTGGTTCATGACATGGCATATAGATATATAAATCTGACAAGAGAAGATATGATTAATTATTATGCCCCACGAATCCAGTTCAAGCAGACACAGAATGTAAGAGAGCCAGGGACATTCAAACTGACATCAAGTATGCTCAGGGCAGAATCAAAAGCAATGCTAGATCTACTCAATAATCATAAAAGTGGTGAGAAGCACGGGGCACAAATAGAAAGTCTAAATATAGCTAGTCATATTGTGCAGTCAGAGTCTGTTAGTTTAATTACAAAGATATTATCTGATCTAGAATTAAACATAACTGAGCCATCAACTCAAGAATATTCTACAACTAAGCACACTTAGGTTGACACAGTGCTGGATAAGTTTTTCCAGAATGAGACACAGAAGTATTTAATAGATGTATTGAAGAAAACAACAGCATGGCATATAGGCCATCTAATAAGGGACATAACAGAAAGCTTGATTGCACATTCAGGATTGAAAAGATCAAAGTATTGGTCCCTACATTCTTATAATAATGGAAATGTTATATTATTTATCCTTCCATCAAAATCACTAGAAGTGGCAGGGTCCTTTATTAGATTTATTACAGTTTTTAGGATAGGACCCGGTCTTGTGGATAAGGACAACCTGGATACTATCTTGATTGATGGTGACTCACAATGGGGAGTATCAAAAGTCATGAGTATTGATTTAAACAGGTTGTTAGCTTTAAACATAGCCTTTGAAAAGGCTTTAATTGCAACAGCCACATGGTTTCAATACTACACAGAAGATCAAGGGCAATTTCCCTTGCAATATGCAATTAGATCTGTCTTTGCAAATCATTTCCTATTAGCTATATGTCAGAAGATGAAACTCTGTGCGATTTTTGATAATTTACGCTATTTAATACCTGCAGTAACATCATTGTATTCTGGTTTTCCTTCATTGATAGAAAAGCTCTTCGAACGGCCCTTTAAGTCTTCATTGGAGGTATATATATATTACAATATAAAAAGCTTATTGGTTGCTCTTGCACAGAATAATAAGGCTAGATTCTATTCTAAGGTAAAGTTGTTGGGTTTGACAGTTGACCAATCAACTGTAGGTGCAAGTGGAGTATATCCTTCTTTCATGTCGCGTATAGTTTATAAACATTACAGAAGTTTGATTTCTGAAGTGACAACCTGTTTCTTCTTATTTGAAAAGGGTCTTCATGGTAATATGAATGAAGAAGCAAAGATTCATCTTGAGACAGTAGAATGGGCACTTAAATTCAGAGAAAAAGAGGAAAAGTATGGAGAATCATTGGTGGAAAATGGATATATGATGTGGGAGCTCAGAGAAAATTCTGATTTGGCTGAGCAACA |
| Yanbian-Aa22s-H3921 | *Apodemus agrarius* | Hantaan virus | L | 1627 | 761–2388 | 99.06–99.87 | ATTCTAAAGAATGGCTGTCCAGAGCTAGAAATTTCTCTTTCTTTGAAGTAAAGGGTACAGCAGTTTTCGATTGCTTTAATTCAAATGAGGCAAATCATTGTCAGAAATATCCCATGTCCCGTAAACCTAGGAACTTTCTGTTAATACAATGTTCCTTAATTACATCTTACAAGCCTGCAACTACTCTTTCAGATCAGATTGATAGCAGAAGGGCTTGTTCGTACATCTTGAATCTAATTCCAGACACACCAGCATCATATTTGGTTCATGACATTGCATATAGATATATAAATCTGACAAGAGAAGATATGATTAATTATTATGCCCCACGAATCCAGTTCAAGCAGACACAGAATGTAAGAGAGCCTGGGACATTCAAACTGACATCAAGTATGCTCAGGGCAGAATCAAAAGCAATGCTAGATCTACTCAATAATCATAAAAGTGGTGAGAAGCACGGGGCACAAATAGAAAGTCTAAATATAGCTAGTCATATTGTGCAGTCAGAGTCTGTTAGTTTAATTACAAAGATATTATCTGATCTAGAATTAAACATAACTGAGCCATCAACTCAAGAATATTCTACAACTAAGCACACTTATGTTGACACAGTGCTGGATAAGTTCTTCCAGAATGAGACACAGAAGTATTTAATAGATGTATTGAAGAAAACAACAGCATGGCATATAGGCCATCTAATAAGGGACATAACAGAAAGCTTGATTGCACATTCAGGATTGAAAAGATCAAAGTATTGGTCCCTACATTCTTATAATAATGGAAATGTTATATTATTTATCCTTCCATCAAAATCACTAGAAGTGGCAGGGTCCTTTATTAGATTTATTACAGTTTTTAGGATAGGACCCGGTCTTGTGGATAAGGACAACCTGGATACTATCTTGATTGATGGTGACTCACAATGGGGAGTATCAAAAGTCATGAGTATTGATTTAAACAGGTTGTTAGCTTTAAACATAGCCTTTGAAAAGGCTTTAATTGCAACAGCCACATGGTTTCAATACTACACAGAAGATCAAGGGCAATTTCCCTTGCAATATGCAATTAGATCTGTCTTTGCAAATCATTTCCTATTAGCTATATGTCAGAAGATGAAACTCTGTGCGATTTTTGATAATTTACGCTATTTAATACCTGCAGTAACATCATTGTATTCTGGTTTTCCTTCATTGATAGAAAAGCTCTTCGAACGGCCCTTTAAGTCTTCATTGGAGGTATATATATATTACAATATAAAAAGCTTATTGGTTGCTCTTGCGCAGAATAATAAGGCTAGATTCTATTCTAAGGTAAAGTTGTTGGGTTTGACAGTTGACCAATCAACTGTAGGTGCAAGTGGAGTATATCCTTCTTTCATGTCGCGTATAGTTTATAAACATTACAGAAGTTTGATTTCTGAAGTGACAACCTGTTTCTTCTTATTTGAAAAGGGTCTTCATGGTAATATGAATGAAGAAGCAAAGATTCATCTTGAGACAGTAGAATGGGCACTTAAATTCAGAGAAAAAGAGGAAAAGTATGGAGAATCATTGGTGGAAAATGGATATATGATGTGGGAGCTCAGAGAAAATTCTGATTTGGCTGAGCAACAACTATACTGTCAGGAT |
| Yanbian-Aa22s-H3924 | *Apodemus agrarius* | Hantaan virus | L | 1635 | 739–2374 | 99.21–99.94 | AATATTGAAGATTTAGTTGATTATTCTAAAGAATGGCTATCCAGAGCTAGAAATTTCTCTTTCTTTGAAGTAAAGGGTACAGCAGTTTTCGATTGCTTTAATTCAAATGAGGCAAATCATTGTCAGAAATATCCCATGTCCCGTAAACCTAGGAACTTTCTGTTAATACAATGTTCCTTAATTACATCTTACAAGCCTGCAACTACTCTTTCAGATCAGATTGATAGCAGAAGGGCTTGTTCGTACATCTTGAATCTAATTCCAGACACACCAGCATCATATTTGGTTCATGACATGGCATATAGATATATAAATCTGACAAGAGAAGATATGATTAATTATTATGCCCCACGAATCCAGTTCAAGCAGACACAGAATGTAAGAGAGCCAGGGACATTCAAACTGACATCAAGTATGCTCAGGGCAGAATCAAAAGCAATGCTAGATCTACTTAATAATCATAAAAGTGGTGAGAAGCACGGGGCACAAATAGAAAGTCTAAATATAGCTAGTCATATTGTGCAGTCAGAGTCTGTTAGTTTAATTACAAAGATATTATCTGATCTAGAATTAAACATAACTGAGCCATCAACTCAAGAATATTCTACAACTAAGCACACTTATGTTGACACAGTGCTGGATAAGTTTTTCCAGAATGAGACACAGAAGTATTTAATAGATGTATTGAAGAAGACAACAGCATGGCATATAGGCCATCTAATAAGGGACATAACAGAAAGCTTGATTGCACATTCAGGATTGAAAAGATCAAAGTATTGGTCCCTACATTCTTATAATAATGGAAATGTTATATTATTTATCCTTCCATCAAAATCACTAGAAGTGGCAGGGTCCTTTATTAGATTTATTACAGTTTTTAGGATAGGACCCGGTCTTGTGGATAAGGACAACCTGGATACTATCTTGATTGATGGTGACTCACAATGGGGAGTATCAAAAGTCATGAGTATTGATTTAAACAGGTTGTTAGCTTTAAACATAGCCTTTGAAAAGGCTTTAATTGCAACAGCCACATGGTTTCAATACTACACAGAAGATCAAGGGCAATTTCCCTTGCAATATGCAATTAGATCTGTCTTTGCAAATCATTTCCTATTAGCTATATGTCAGAAGATGAAACTCTGTGCGATTTTTGATAATTTGCGCTATTTAATACCTGCAGTAACATCATTGTATTCTGGTTTTCCTTCATTGATAGAAAAGCTCTTCGAACGGCCCTTTAAGTCTTCATTGGAGGTATATATATATTACAATATAAAAAGCTTATTGGTTGCTCTTGCACAGAATAATAAGGCTAGATTCTATTCTAAGGTAAAGTTGTTGGGTTTGACAGTTGACCAATCAACTGTAGGTGCAAGTGGAGTATATCCTTCTTTCATGTCGCGTATAGTTTATAAACATTACAGAAGTTTGATTTCTGAAGTGACAACCTGTTTCTTCTTATTTGAAAAGGGTCTTCATGGTAATATGAATGAAGAAGCAAAGATTCATCTTGAGACAGTAGAATGGGCACTTAAATTCAGAGAAAAAGAGGAAAAGTATGGAGAATCATTGGTGGAAAATGGATATATGATGTGGGAGCTCAGAGAAAATTCTGATTTGGCTGAGCAACAAC |
| Yanbian-Aa22s-H3930 | *Apodemus agrarius* | Hantaan virus | L | 1795 | 777–2572 | 99.28–99.94 | ATCCAGAGCTAGAAATTTCTCTTTCTTTGAAGTAAAGGGTACAGCAGTTTTCGATTGCTTTAATTCAAATGAGGCAAATCATTGTCAGAAATATCCCATGTCCCGTAAACCTAGGAACTTTCTGTTAATACAATGTTCCTTAATTACATCTTACAAGCCTGCAACTACTCTTTCAGATCAGATTGATAGCAGAAGGGCTTGTTCGTACATCTTGAATCTAATTCCAGACACACCAGCATCATATTTGGTTCATGACATGGCATATAGATATATAAATCTGACAAGAGAAGATATGATTAATTATTATGCCCCACGAATCCAGTTCAAGCAGACACAGAATGTAAGAGAGCCAGGGACATTCAAACTGACATCAAGTATGCTCAGGGCAGAATCAAAAGCAATGCTAGATCTACTTAATAATCATAAAAGTGGTGAGAAGCACGGGGCACAAATAGAAAGTCTAAATATAGCTAGTCATATTGTGCAGTCAGAGTCTGTTAGTTTAATTACAAAGATATTATCTGATCTAGAATTAAACATAACTGAGCCATCAACTCAAGAATATTCTACAACTAAGCACACTTATGTTGACACAGTGCTGGATAAGTTTTTCCAGAATGAGACACAGAAGTATTTAATAGATGTATTGAAGAAGACAACAGCATGGCATATAGGCCATCTAATAAGGGACATAACAGAAAGCTTGATTGCACATTCAGGATTGAAAAGATCAAAGTATTGGTCCCTACATTCTTATAATAATGGAAATGTTATATTATTTATCCTTCCATCAAAATCACTAGAAGTGGCAGGGTCCTTTATTAGATTTATTACAGTTTTTAGGATAGGACCCGGTCTTGTGGATAAGGACAACCTGGATACTATCTTGATTGATGGTGACTCACAATGGGGAGTATCAAAAGTCATGAGTATTGATTTAAACAGGTTGTTAGCTTTAAACATAGCCTTTGAAAAGGCTTTAATTGCAACAGCCACATGGTTTCAATACTACACAGAAGATCAAGGGCAATTTCCCTTGCAATATGCAATTAGATCTGTCTTTGCAAATCATTTCCTATTAGCTATATGTCAGAAGATGAAACTCTGTGCGATTTTTGATAATTTGCGCTATTTAATACCTGCAGTAACATCATTGTATTCTGGTTTTCCTTCATTGATAGAAAAGCTCTTCGAACGGCCCTTTAAGTCTTCATTGGAGGTATATATATATTACAATATAAAAAGCTTATTGGTTGCTCTTGCACAGAATAATAAGGCTAGATTCTATTCTAAGGTAAAGTTGTTGGGTTTGACAGTTGACCAATCAACTGTAGGTGCAAGTGGAGTATATCCTTCTTTCATGTCGCGTATAGTTTATAAACATTACAGAAGTTTGATTTCTGAAGTGACAACCTGTTTCTTCTTATTTGAAAAGGGTCTTCATGGTAATATGAATGAAGAAGCAAAGATTCATCTTGAGACAGTAGAATGGGCACTTAAATTCAGAGAAAAAGAGGAAAAGTATGGAGAATCATTGGTGGAAAATGGATATATGATGTGGGAGCTCAGAGAAAATTCTGATTTGGCTGAGCAACAACTATACTGTCAGGATGCTGTAGAGTTAGCAGCAATAGAATTGAACAAAGTTTTATCTACAAAATCCAGTGTTGTAGCTAACAGTATATTAAATAAGAATTGGGAAGAACCATATTTTAGTCAAACAAGAAATATTAGTTTAAAAGGTATGTCCGGGCAGGTTCAAGAGGATGGCCACCTCTCATCATCTGTAACAATTA |
| Yanbian-Aa22s-H3933 | *Apodemus agrarius* | Hantaan virus | L | 1714 | 792–2506 | 99.24–99.88 | TTTCTCTTTCTTTGAAGTAAAGGGTACAGCAGTTTTCGATTGCTTTAATTCAAATGAGGCAAATCATTGTCAGAAATATCCCATGTCCCGTAAACCTAGGAACTTTCTGTTAATACAATGTTCCTTAATTACATCTTACAAGCCTGCAACTACTCTTTCAGATCAGATTGATAGCAGAAGGGCTTGTTCGTACATCTTGAATCTAATTCCAGACACACCAGCATCATATTTGGTTCATGACATGGCATATAGATATATAAATCTGACAAGAGAAGATATGATTAATTATTATGCCCCACGAATCCAGTTCAAGCAGACACAGAATGTAAGAGAGCCTGGGACATTCAAACTGACATCAAGTATGCTCAGGGCAGAATCAAAAGCAATGCTAGATCTACTCAATAATCATAAAAGTGGTGAAAAGCACGGGGCACAAATAGAAAGTCTAAATATAGCTAGTCATATTGTGCAGTCAGAGTCTGTTAGTTTAATTACAAAGATATTATCTGATCTAGAATTAAACATAACTGAGCCATCAACTCAAGAATATTCTACAACTAAGCACACTTATGTTGACACAGTGCTGGATAAGTTTTTTCAGAATGAGACACAGAAGTATTTAATAGATGTATTGAAGAAGACAACAGCATGGCATATAGGCCATCTAATAAGGGACATAACAGAAAGCTTGATTGCACATTCAGGATTGAAAAGATCAAAGTATTGGTCCCTACATTCTTATAATAATGGAAATGTTATATTATTTATCCTTCCATCAAAATCACTAGAAGTGGCAGGGTCCTTTATTAGATTTATTACAGTTTTTAGGATAGGACCCGGTCTTGTGGATAAGGACAACCTGGATACTATCTTGATTGATGGTGACTCACAATGGGGAGTATCAAAAGTCATGAGTATTGATTTAAACAGGTTGTTAGCTTTAAACATAGCCTTTGAAAAGGCTTTAATTGCAACAGCCACATGGTTTCAATACTACACAGAAGATCAAGGGCAATTTCCCTTGCAATATGCAATTAGATCTGTCTTTGCAAATCATTTCCTATTAGCTATATGTCAGAAGATGAAACTCTGTGCGATTTTTGATAATTTACGCTATTTAATACCTGCAGTAACATCATTGTATTCTGGTTTTCCTTCATTGATAGAAAAGCTCTTCGAACGGCCCTTTAAGTCTTCATTGGAGGTATATATATATTACAATATAAAAAGCTTATTGGTTGCTCTTGCACAGAATAATAAGGCTAGATTCTATTCAAAGGTAAAGTTGTTGGGTTTGACAGTTGACCAATCAACTGTAGGTGCAAGTGGAGTATATCCTTCTTTCATGTCGCGTATAGTTTATAAACATTACAGAAGTTTGATTTCTGAAGTGACAACCTGTTTCTTCTTATTTGAAAAGGGTCTTCATGGTAATATGAATGAAGAAGCAAAGATTCATCTTGAGACAGTAGAATGGGCACTTAAATTCAGAGAAAAAGAGGAAAAGTATGGAGAATCATTGGTGGAAAATGGATATATGATGTGGGAGCTCAGAGAAAATTCTGATTTGGCTGAGCAACAACTATACTGTCAGGATGCTGTAGAGTTAGCAGCAATAGAATTGAACAAAGTTTTATCTACAAAATCCAGTGTTGTAGCTAACAGTATATTAAATAAGAATTGGGAAGAACCATATTTTAGTCAAACAAGAAATA |
| Yanbian-Aa22a-H3995 | *Apodemus agrarius* | Amur virus | L | 576 | 121–697 | 99.31 | CAGTAAGGCATGACATTGTGGACCAGATGATAAAACATGACTGGTCTGATAACAAAGACTCAGAAGAGCCAATAGGTAAAGTGTTATTGTTTGCAGGTGTTCCCTCAAACATTATAACAGCACTAGAGAAGAAGATTATTCCAAACCATCCCACAGGGAAAAATCTTAAAGCATTCTTTAAAATGACACCTGACAACTATAAGATTACTGGAACTACCATAGAGTTTGTTGAGGTGACTGTCACAGCAGATGTGGACAAAGGCATAAGAGAAAAAAAGATTAAGTATGAGGCAGGATTAGCCTATATTGAACAGGAACTTCATAAGTTCTATTTGAAAGGTGAGATTCCACAACCATATAAGATTACATTGAATGTTGTTGCAGTTCGGACTGATGGGTCTAATATCACAACTCAATGGCCTAGTAGAAGGAACGACGGTGTTGTCCAGTATATGAGATTAGTGCAGGCTGAGATAAGTTATGTTAGAGAGCACTTAATCAAACCAGATGAAAGGGCTGCTTTAGAGGCAATGTTTAATTTGAAGTTCAATATAAGTACATATAAAAGCCAGCCTTA |
| Yanbian-Aa22s-H3932 | *Apodemus agrarius* | Amur virus | M | 357 | 890–1247 | 99.16 | TCATACTCCATTGTAGGGCCTGCCAATGCAAAAGTACCCCATAGTGCCAGTGCAGATACACTGAGTTTAGTTGCCTTTTCAGGGATCCCATCCTACTCGTCTTTCAGTGTCCTCACAGGATCTACAGATGCCCGCCATGTCTTTAGCCCAGGTTTCTTTCCACAACTAAACCAGACAAGGTGTGATAAAACTGCCATACCATTAACCTGGACAGGAATGATTGATCTACCTGGTTATTATGAAGCAGTACACCCTTGCACTGTCTTCTGTGTTTTGTCCGGTCCTGGCGCGTCCTGTGAAGCATTTTCAGAAGGGGGG-ATCTTTAATATAACTTCTCCCATGTGTTTAGTTTCAAAG |
| Yanbian-Aa22s-H3939 | *Apodemus agrarius* | Amur virus | L | 483 | 1717–2200 | 99.59 | AACAGGCTACTGGCATTAAATATTGCATTTGAGAAGGCTTTAATTGCAACAGCCACATGGTTTCAGTATTATACTGAAGATCAGGGACAATTTCCTCTACAATATGCAATAAGATCAGTGTTTGCAAATCATTTCCTTTTGTCAGTTTGCCAAAAGATGAAACTTTGTGCAATTTTTGATAATTTACGGTACCTTATCCCTGCTGTCACATCTTTATACTCAGGATTCCCTTCCTTAATAGAAAAACTATTTGAGAGACCGTTTAAATCAGCATTAGAAGTCTATGTGTATTATAATATAAAAAGTCTATTAGTGGCTCTGGCACAAAACAATAAGGCACCGTTTTACTCAAAAGTCAAATTGTTAGGGTTAACTGTAGATCAGTCAACTGTAGGTGCAAGCGGAATTTATCCTTCATTTATGTCACGTATAGTTTACAAACACTATAGAAGTTTAATTTCTGAAGTGACAACATGTTTCTTCC |
| Yanbian-Aa23s-H4157 | *Apodemus agrarius* | Amur virus | L | 506 | 1694–2200 | 99.4 | CAAAAGTTATGAGTATTGATTTGAACAGGTTACTGGCATTAAATATTGCATTTGAGAAGGCTTTAATTGCAACAGCCACATGGTTTCAGTATTATACTGAAGATCAGGGACAATTTCCTCTACAATATGCAATAAGATCAGTGTTTGCAAATCATTTCCTTTTGTCAGTTTGCCAAAAGATGAAACTTTGTGCAATTTTTGATAATTTACGGTACCTTATCCCTGCTGTCACATCTTTATACTCAGGATTCCCTTCCTTAATAGAAAAACTATTTGAGAGACCGTTTAAATCAGCATTAGAAGTCTATGTGTATTATAATATAAAAAGTCTATTAGTGGCTCTGGCACAAAACAATAAGGCACCGTTTTACTCAAAAGTCAAATTGTTAGGGTTAACTGTAGATCAGTCAACTGTAGGTGCAAGCGGAATTTATCCTTCATTTATGTCACGTATAGTTTACAAACACTATAGAAGTTTAATTTCTGAAGTGACAACATGTTTCTTCC |

Positions are relative to the corresponding complete genome sequences obtained in this study. The percentage of nucleotide sequence identity is compared to the respective complete genome.
